# OCA7 is a melanosome membrane protein that defines pigmentation by regulating early stages of melanosome biogenesis

**DOI:** 10.1016/j.jbc.2022.102669

**Published:** 2022-11-09

**Authors:** Wyatt C. Beyers, Anna M. Detry, Santiago M. Di Pietro

**Affiliations:** Department of Biochemistry and Molecular Biology, Colorado State University, Fort Collins, Colorado, USA

**Keywords:** organelle, melanogenesis, membrane protein, Rab, confocal microscopy, CRISPR/Cas, oculocutaneous albinism type 7, melanosome biogenesis, membrane traffic, BFP2, blue fluorescent protein 2, CAF, core amyloid fragment, Cas9, CRISPR-associated protein 9, cDNA, complementary DNA, crRNA, CRISPR RNA, EGFP, enhanced GFP, FRAP, fluorescence recovery after photobleaching, HA, hemagglutinin, IB, immunoblot, IgG, immunoglobulin G, IP, immunoprecipitation, iRFP, infrared fluorescent protein, IS, Interswitch, LRR, leucine-rich repeat, M/C, membrane and cytosol, MELOPS, MElanosome LOcalized pH Sensor, NA, numerical aperture, OCA, oculocutaneous albinism, OCA7, oculocutaneous albinism type 7, PMEL, premelanosome protein, RIPA, radioimmunoprecipitation assay, ROI, region of interest, S1, switch 1, S2, switch 2, SD -L-W, synthetic dropout media lacking leucine and tryptophan, SR, super resolution, TBST, Tris-buffered saline with Tween-20, tracrRNA, transactivating CRISPR RNA, TX100, Triton X-100, Y2H, yeast two hybrid

## Abstract

Mutations in C10orf11 (oculocutaneous albinism type 7 [OCA7]) cause OCA, a disorder that presents with hypopigmentation in skin, eyes, and hair. The OCA7 pathophysiology is unknown, and there is virtually no information on the OCA7 protein and its cellular function. Here, we discover that OCA7 localizes to the limiting membrane of melanosomes, the specialized pigment cell organelles where melanin is synthesized. We demonstrate that OCA7 is recruited through interaction with a canonical effector–binding surface of melanosome proteins Rab32 and Rab38. Using newly generated OCA7-KO MNT1 cells, we show OCA7 regulates overall melanin levels in a melanocyte autonomous manner by controlling melanosome maturation. Importantly, we found that OCA7 regulates premelanosome protein (PMEL) processing, impacting fibrillation and the striations that define transition from melanosome stage I to stage II. Furthermore, the melanosome lumen of OCA7-KO cells displays lower pH than control cells. Together, our results reveal that OCA7 regulates pigmentation through two well-established determinants of melanosome biogenesis and function, PMEL processing, and organelle pH.

Oculocutaneous albinism (OCA) is a disorder displaying loss of melanin pigment in the skin, eyes, and hair. OCA patients have heightened susceptibility to skin cancer and poor visual acuity (1, 2, 3, 4, 5). Eight forms of OCA exist, and the corresponding genes and protein functions have been elucidated for most of them (4, 6, 7, 8, 9, 10, 11, 12, 13, 14, 15, 16). However, while mutations in OCA type 7 (OCA7) (a.k.a. C10orf11 and LRMDA) were identified in OCA patients and OCA7 SNPs were associated with eyebrow color variations in genome-wide association study analysis, the cellular function of OCA7 and corresponding disease mechanism are unknown (17).

Melanin synthesis occurs in specialized lysosome-related organelles called melanosomes, which are classified as stage I, II, III, or IV largely based on their appearance by electron microscopy. Melanosome biogenesis starts with stage I melanosomes that resemble multivesicular endosomes, contain the premelanosome protein (PMEL), and often present an electron dense coat of clathrin (18). Early stage melanosomes remodel their membranes in a PIKfyve-dependent manner and generate intraluminal vesicles in a process involving CD63 (19, 20). An amyloid sequestering protein, apolipoprotein E, is present on the surface of melanosome intraluminal vesicles where it forms a cylindrical structure that functions as a seed for PMEL association and fibrillation (21). PMEL is proteolytically processed, associates with the intraluminal vesicles, and forms functional amyloid fibrils that work to package melanin as it is made (22). Melanosomes containing mature PMEL fibrils show striations by electron microscopy and are classified as stage II. Subsequent delivery of tyrosinase family proteins results in melanin synthesis, and pigment begins depositing onto PMEL fibrils, thus defining stage III melanosomes. Additional melanin synthesis and deposition results in more fully pigmented stage IV melanosomes. Despite this detailed morphological characterization, melanosome biogenesis is not fully understood, particularly with regard to regulation of early stages of maturation.

**Figure 10:**
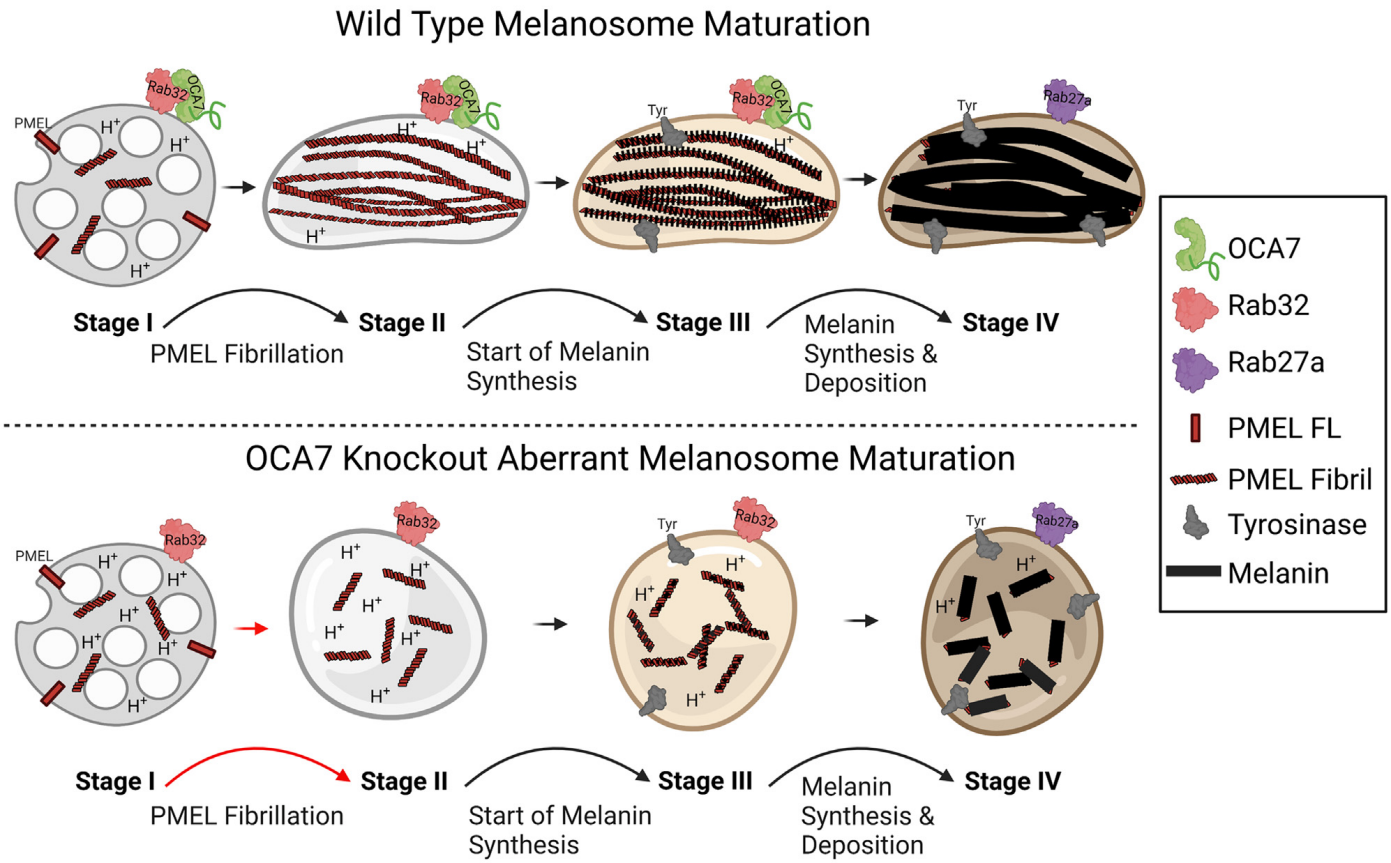
***Cartoon* depiction of melanosome biogenesis defects in OCA7 mutant in melanocytes.** In WT melanocytes, OCA7 is present on melanosome membranes through interaction with Rab32 (and Rab38) that requires the OCA7 LRR domain and Rab32 Interswitch and switch 2 regions. Melanosome biogenesis starts with stage I melanosomes that resemble multivesicular endosomes defined by the presence of full-length and partially processed PMEL protein. As full-length PMEL is processed, it forms the Mα fragment that then associates with the intraluminal vesicles, eventually undergoing additional proteolytic processing and self-association into a mature insoluble fibril, and the melanosome is defined as stage II. Once fibrils are formed, tyrosinase arrives to the melanosome and begins melanin synthesis, and melanin deposits onto PMEL fibrils, defining stage III melanosomes. Additional melanin synthesis results on fully pigmented stage IV melanosomes, which are primarily labeled by Rab27a and not Rab32 and OCA7. In OCA7-KO melanocytes, melanosomes accumulate the PMEL Mα fragment but do not productively form ordered fibrils. Tyrosinase proceeds to make melanin but pigment cannot organize correctly in the absence of normal PMEL fibrils, leading to aberrant stage IV melanosomes seen by electron microscopy. A lower luminal pH in OCA7 deficient melanosomes results in lower tyrosinase activity and decreased melanin synthesis and potentially contributes to PMEL processing defects. The *cartoon* was created with Biorender.com. LRR, leucine-rich repeat; OCA7, oculocutaneous albinism type 7; PMEL, premelanosome protein.

Here, we reveal that OCA7 functions as a melanosome-associated peripheral membrane protein in human melanocytes, likely recruited through interaction with Rab32 and Rab38. OCA7-KO MNT1 melanocytes obtained by CRISPR–CRISPR-associated protein 9 (Cas9) have reduced melanin content, a defect that was rescued by transient expression of OCA7-enhanced GFP (EGFP). The PMEL protein is abnormally processed in OCA7-KO cells and forms fewer fibril striations in melanosomes. The melanosome lumen pH, which regulates melanogenic enzyme activity, is lower in OCA7-KO cells. These findings reveal that OCA7 regulates melanosome function starting at early stages of the biogenesis process.

## Results

### OCA7 localizes to melanosome membranes

**Figure 1:**
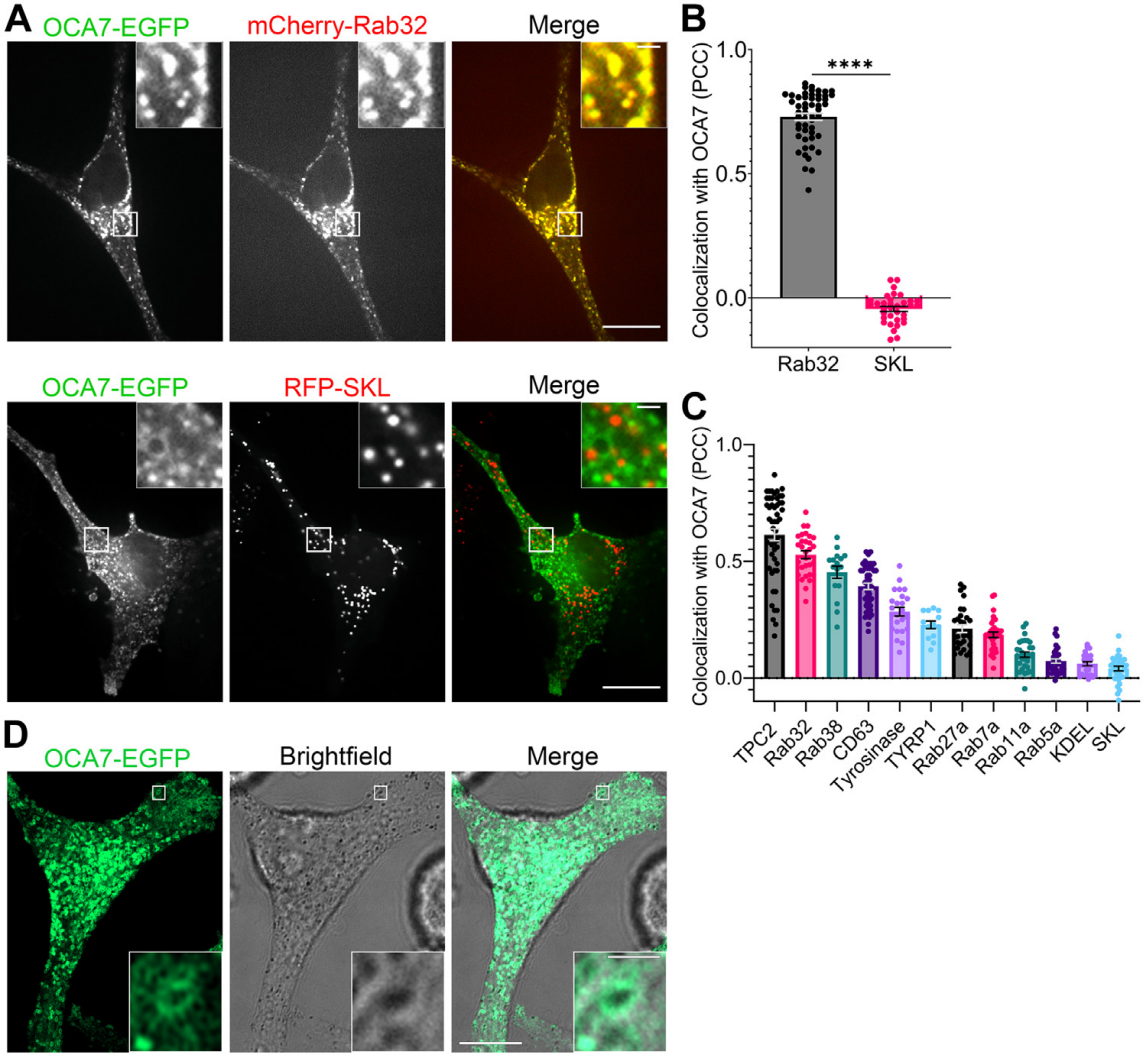
**OCA7 localizes to melanosomes**. *A*, spinning disc confocal fluorescence microscopy analysis of live primary human melanocytes coexpressing OCA7-EGFP with mCherry-Rab32 or RFP-SKL. The scale bars indicate 10 μm for *unmagnified images* and 1 μm for *magnified insets*. *B*, Pearson correlation coefficient (PCC) for images from (*A*). Error bars represent the 95% confidence interval (mCherry-Rab32, n = 55 cells; RFP-SKL, n = 34 cells). *C*, colocalization quantification as determined by PCC for images of MNT1 cells expressing OCA7-EGFP with organelle markers from Fig. S1, showing median with error bars representing the 95% confidence interval. n = 50, n = 30, n = 16, n = 45, n = 24, n = 13, n = 29, n = 33, n = 31, n = 33, n = 25, and n = 32 cells for TPC2-iRFP, mCherry-Rab32, mCherry-Rab38, mCherry-CD63, tyrosinase-mCherry, tyrosinase-P1-mCherry, mCherry-Rab27a, mCherry-Rab7a, mCherry-Rab11a, mCherry-Rab5a, BFP2-KDEL, and RFP-SKL, respectively. *D*, live cell Airyscan super-resolution and brightfield images of MNT1 melanocytes expressing OCA7-EGFP showing a cohort of OCA7 localizes to membranes surrounding melanin pigment. The scale bars indicate 10 μm for *unmagnified image* and 1 μm for *magnified inset*. EGFP, enhanced GFP; iRFP, infrared fluorescent protein; OCA7, oculocutaneous albinism type 7; RFP, red fluorescent protein.

TPC2 is a melanosome-localized cation channel (23, 24). To identify novel proteins on the melanosome membrane, we performed a proximity biotinylation mass spectrometry screen using MNT1 cells expressing TPC2-BioID2. Cells expressing endosome-localized TPC1-BioID2 were analyzed in parallel as a control. Details of this screening will be reported separately. Surprisingly, we detected endogenous OCA7 specifically in proximity to TPC2-BioID2, providing the first molecular evidence that OCA7 may function at melanosomes. Immunofluorescence experiments with available antibodies for endogenous OCA7 were suspect, resulting in nuclear and random cytoplasmic signal. Therefore, we turned to live cell imaging of exogenously expressed OCA7-EGFP to visualize its localization. Attempts to amplify two potential transcript variants of OCA7 encoding a 226 or 198 amino acid protein from MNT1 complementary DNA (cDNA) were only successful for the first, suggesting it is the predominant variant. Live cell confocal fluorescence microscopy of OCA7-EGFP expressed in primary human melanocytes showed discrete cytoplasmic puncta that colocalized with melanosome marker mCherry-Rab32 but not peroxisome marker RFP-SKL as quantified by Pearson correlation coefficient (Fig. 1, *A* and *B*). Consistently, OCA7-EGFP colocalized highly with melanosome markers TPC2-infrared fluorescent protein (iRFP), mCherry-Rab32, mCherry-Rab38, and mCherry-CD63 in MNT1 cells, a well-validated system to study melanosome biology (representative images shown in Fig. S1 and quantification in Fig. 1*C*). OCA7-EGFP colocalized to a lesser extent with mature melanosome markers, tyrosinase-mCherry, tyrosinase-p1-mCherry, and mCherry-Rab27a (Figs. S1 and 1*C*). OCA7-EGFP showed low colocalization with endosomal proteins mCherry-Rab7a, mCherry-Rab11a, and mCherry-Rab5a and negligible colocalization with endoplasmic reticulum and peroxisome markers blue fluorescent protein 2 (BFP2)-KDEL and RFP-SKL (Figs. S1 and 1*C*). Airyscan super-resolution (SR) images of OCA7-EGFP overlayed with brightfield images show a small proportion of OCA7-EGFP decorating pigmented melanosomes (Fig. 1*D*). The microscopy data confirm OCA7-EGFP localization to melanosomes and suggest an enrichment in less mature melanosomes. During OCA7 localization studies, we observed that fixation of MNT1 cells resulted in loss of OCA7-EGFP puncta, leaving only diffuse OCA7-EGFP signal, likely explaining why immunofluorescence experiments are unreliable. While overexpressed OCA7-EGFP clearly colocalizes with melanosome markers, it is conceivable that endogenously expressed tagless OCA7 may have additional cellular localizations or context-dependent localization that we were unable to detect.

### OCA7 interacts physically with melanosome biogenesis proteins Rab32 and Rab38

**Figure 2:**
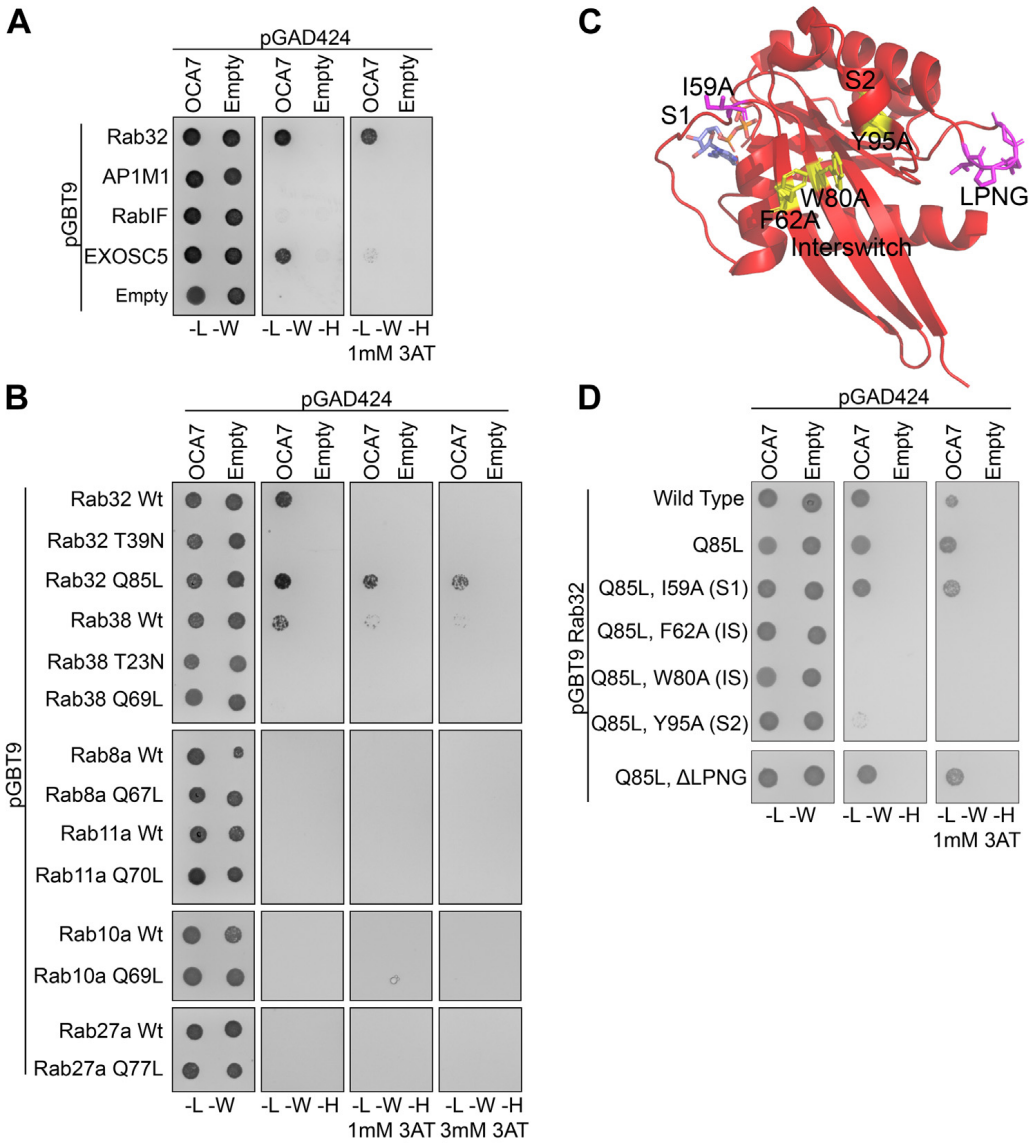
**OCA7 interacts with Rab32 and Rab38 through the Interswitch (IS) and switch 2 (S2) regions.***A*, Y2H assay showing OCA7 reactivity with Rab32 and EXOSC5 but not AP1M1 or RabIF. Y2H shown is representative of three experiments. *B*, Y2H assay showing specific interactions between OCA7 and melanosome membrane proteins, Rab32 and Rab38, but not other melanosome or endosome Rabs, Rab8a, Rab10a, Rab11a, or Rab27a or their constitutively active mutants. Y2H shown is representative of two experiments. *C*, crystal structure of Rab32 GTP (Protein Data Bank code: 6FF8) highlighting switch 1 (S1), IS, S2, and LPNG loop of Rab32 mutated in the Y2H in (*D*). Residues dispensable for OCA7 reactivity are indicated in *magenta*, and residues that influence reactivity are highlighted in *yellow* (31). *D*, Y2H assay to test for Rab32 residues necessary for OCA7 interaction. Rab32 constitutively active mutant (Q85L) was subsequently mutated in S1 (I59A), IS (F62A or W80A), S2 (Y95A), or LPNG loop regions to map binding to OCA7. Y2H shown is representative of two experiments. For each Y2H, growth on +histidine plates indicates successful cotransformation with pGBT9 and pGAD424 vectors. Growth on −histidine plates indicates interaction between the bait and prey proteins, and growth on plates containing 3-amino-1,2,4-triazole (3AT) indicates stronger interactions. The T/N mutation prevents Rabs from exchanging GDP for GTP, keeping it in the “off” conformation. The Q/L mutation prevents Rabs from hydrolyzing GTP, keeping it in the “on” conformation. OCA7, oculocutaneous albinism type 7; Y2H, yeast two hybrid.

The Human Reference Interactome database listed interactions between OCA7 and Rab32, AP1M1, RabIF, and EXOSC5 based on unbiased proteome wide yeast two hybrid (Y2H) data (25). We reproduced the positive Y2H result with Rab32 or EXOSC5 but not with AP1M1 or RabIF (Fig. 2*A*). Rab32 resides on melanosome membranes and is involved in mediating membrane trafficking events during melanosome biogenesis through interaction with various effectors, making it an obvious candidate for a functional interaction with OCA7 (26, 27, 28). In contrast, EXOSC5 is a component of a ribonuclease complex in nucleoli, thus it is unlikely to function with OCA7 at melanosomes (29, 30). Rab proteins are thought of as molecular switches because in their GTP-bound state, they adopt an “on” conformation and recruit effector proteins to specific organelles. Upon GTP hydrolysis, Rabs switch to the “off” conformation and cease effector recruitment. Mutagenesis of highly conserved residues in Rab GTPases can be exploited to control which nucleotide is bound, a strategy that has been used in previous studies to control whether Rabs, including Rab32 and Rab38, are “on” or “off” (27, 28, 31). We expanded the Y2H analysis to test for interactions between OCA7 and WT or constitutively active mutants (GTP locked, Q-to-L mutation) of several endosomal or melanosomal Rabs. Dominant negative forms (GDP locked, T-to-N mutation) were also tested for Rab32 and Rab38. Results confirmed OCA7 reactivity toward Rab32, with a preference for the constitutively active mutant Q85L over WT or dominant negative mutant T39N. OCA7 also interacted with Rab38 WT, which is closely related to Rab32, but not with other Rabs tested (Fig. 2*B*). The data suggest that OCA7 interacts specifically with melanosomal Rab proteins involved in melanosome biogenesis. Rab32 has canonical switch 1 (S1), Interswitch (IS), switch 2 (S2) regions and a family specific LPNG loop that, based on crystal structure information and the high conservation among Rab proteins, are predicted to form the binding interface for effector protein interactions (Fig. 2*C*) (31, 32). In addition, previous studies concerning Rab32 and Rab38 demonstrated that point mutagenesis of residues in Switch regions can disrupt binding to Varp (VPS9-ankyrin-repeat protein) and myosin 5C (27, 31, 33). The same point mutant forms of Rab32 were used here to test for their effect on interaction with OCA7 (Fig. 2*C*). Mutation of S1 (I59A) or the LPNG loop did not impact interaction with OCA7, but mutation of IS (F62A or W80A) or S2 (Y95A) disrupted OCA7 binding, suggesting OCA7 interacts with Rab32 at a canonical Rab effector–binding interface involving IS and S2 regions (Fig. 2*D*).

### OCA7 is peripherally associated with melanosome membranes

**Figure 3:**
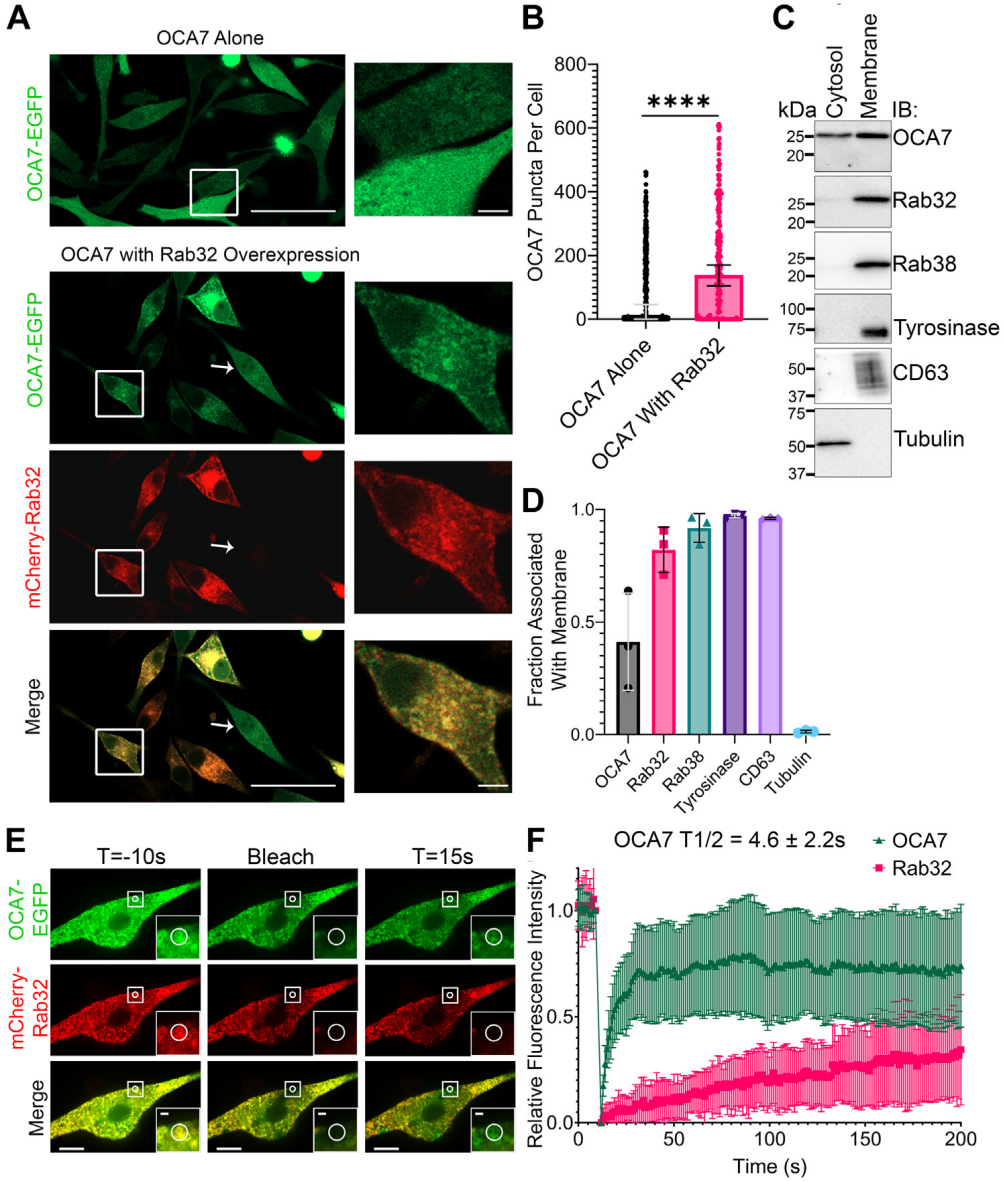
**OCA7 is peripherally associated with melanosome membranes.***A*, live cell Airyscan SR-4Y confocal fluorescence images of MNT1 melanocytes expressing OCA7-EGFP with or without coexpression of mCherry-Rab32. *Magnified insets* show areas of cells where OCA7-EGFP has high cytosolic background (OCA7 alone) or is highly punctate (OCA7 with Rab32 overexpression). In addition, the *white arrow* shows a cell expressing a high level of OCA7-EGFP but not mCherry-Rab32, noting its more diffuse OCA7-GFP distribution compared with adjacent cells that cooverexpress mCherry-Rab32. The scale bars indicate 50 μm for *unmagnified image* and 5 μm for *magnified inset*. *B*, quantification of OCA7-EGFP puncta per cell as determined by single-particle analysis using Zeiss Zen Blue software for images depicted in (*A*). The graph shows the median with error bars representing the 95% confidence interval. Melanosomes were counted in n = 370 and n = 202 cells for OCA7-EGFP alone or OCA7-EGFP with mCherry-Rab32, respectively. Medians were compared using the Mann–Whitney test. ∗∗∗∗ corresponds *p* < 0.0001. *C*, immunoblotting analysis of membrane and cytosol fractions from MNT1 cell homogenate separated by ultracentrifugation and probed with the indicated antibodies for endogenous proteins. *D*, quantification of (*C*) showing the fraction of each protein in the cytosol and membrane fractions. The graph shows mean ± SD. Three independent experiments were quantified. *E*, FRAP experiment with MNT1 cells expressing OCA7-EGFP and mCherry-Rab32. Images are shown for time points 10 s before photobleaching, immediately after, and 15 s after. *Circles* in the *insets* indicate melanosomes analyzed by FRAP. The scale bars indicate 10 μm for *unmagnified images* and 1 μm for *magnified insets*. *F*, quantification of FRAP time lapses shown in (*E*) depicting mean fluorescence signal relative to the time point immediately before photobleaching. Error bars represent SD (200 time points were measured for n = 31 melanosomes imaged in 12 cells). OCA7 T1/2 is the mean ± SD. EGFP, enhanced GFP; FRAP, fluorescence recovery after photobleaching; OCA7, oculocutaneous albinism type 7; SR, su–per resolution.

OCA7-EGFP localizes to not only melanosomes but also diffusely in the cytosol, particularly in cells with higher expression levels. Intriguingly, highly expressed OCA7-EGFP displayed a more punctate distribution if mCherry-Rab32 was also overexpressed in the same cell (Fig. 3, *A* and *B*). OCA7 has no predicted transmembrane or membrane-interacting domain, suggesting OCA7 is a peripheral membrane protein recruited through interaction with proteins on the melanosome surface, such as Rab32. Fractionation of MNT1 cell homogenates by ultracentrifugation followed by immunoblot (IB) analysis revealed endogenous OCA7 is divided almost evenly between membrane and cytosol (M/C) fractions, suggesting it is weakly or dynamically associated with membranes (Fig. 3, *C* and *D*). In contrast, integral membrane proteins, tyrosinase and CD63, or lipidated membrane proteins, Rab32 and Rab38, were highly enriched in the membrane fraction (Fig. 3, *C* and *D*). Fluorescence recovery after photobleaching (FRAP) experiments revealed that OCA7-EGFP fluorescence at melanosomes recovered in a matter of seconds (T1/2 = 4.6 ± 2.2 s), whereas mCherry-Rab32 and mCherry-CD63 did not reach half recovery in the time frame of the assay (Figs. 3, *E* and *F* and S2). Together, the data suggest that OCA7 is peripherally associated with melanosome membranes where it is dynamically recruited potentially by Rab32/Rab38 and subjected to rapid turnover.

### Rab32 or Rab38 membrane localization is sufficient for OCA7 recruitment to membranes

**Figure 4:**
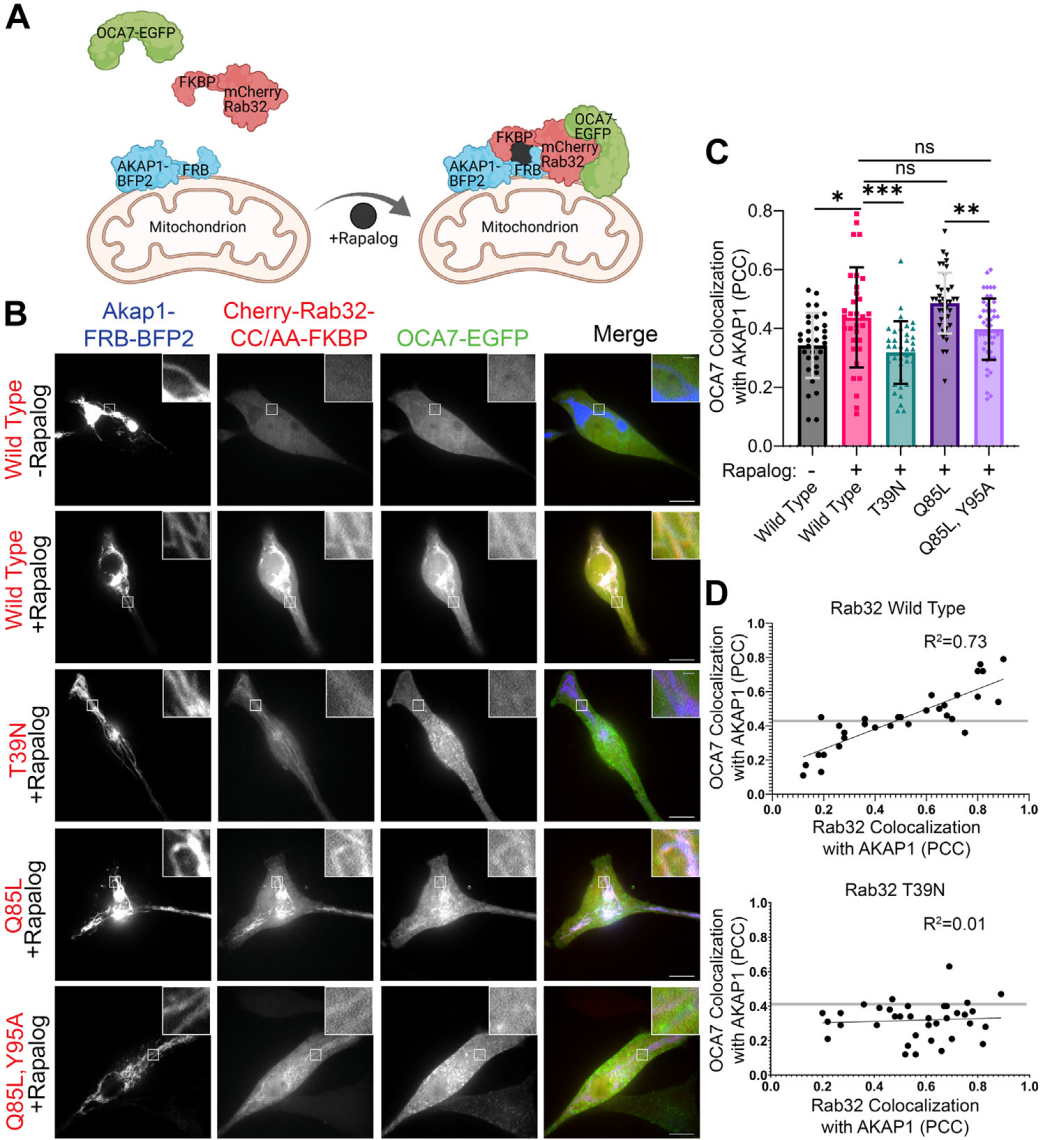
**Rab32 membrane localization is sufficient for OCA7 recruitment to membranes.***A*, *cartoon* depicting Rab32 mislocalization assay. The *cartoon* was created with Biorender.com. *B*, spinning disc confocal fluorescence microscopy images of MNT1 cells expressing OCA7-EGFP, AKAP1-FRB-BFP2, and mCherry-Rab32-CC/AA-FKBP (WT or various mutants) in the absence or presence of 100 nM rapalog. In the absence of rapalog, mCherry-Rab32-CC/AA-FKBP has a cytosolic distribution and OCA7-EGFP has a cytosolic or punctate (melanosome) distribution. Upon addition of 100 nM rapalog, mCherry-Rab32-CC/AA-FKBP (WT and various mutants) relocalize to AKAP1-FRB-BFP2–labeled mitochondria. Concomitantly, OCA7-EGFP mislocalizes to mitochondria with WT or GTP-locked, constitutively active mutant (Q85L) mCherry-Rab32-CC/AA-FKBP but not the GDP-locked, dominant negative mutant (T39N). Expression of the corresponding Rab32 Q85L,Y95A double mutant (Q85L,Y95A), which is constitutively active but unable to bind OCA7 shows reduced OCA7-EGFP recruitment to mitochondria compared with the Rab32 Q85L constitutively active mutant. *Insets* show regions where mitochondria are visible (AKAP1-FRB-BFP2 channel). The scale bars indicate 10 μm for *unmagnified images* and 1 μm for *magnified insets*. *C*, graph of Pearson correlation coefficient for images in (*B*) depicting OCA7-EGFP colocalization with AKAP1-FRB-BFP2, indicating mislocalization to mitochondria. The graph shows mean ± SD. n = 32, n = 32, n = 36, n = 40, and n = 48 cells were analyzed for WT −rapalog, WT +rapalog, T39N +rapalog, Q85L +rapalog, and Q85L,Y95A +rapalog, respectively. Statistical significance was tested by one-way ANOVA and post hoc Tukey tests. For statistical comparisons, ns, ∗, ∗∗, and ∗∗∗ correspond to *p* > 0.05, *p* < 0.05, *p* < 0.01, and *p* < 0.001, respectively. *D*, regression analysis investigating the correlation between mCherry-Rab32-CC/AA-FKBP/AKAP1-FRB-BFP2 dimerization efficiency (Rab32 colocalization with AKAP1) and OCA7-EGFP recruitment to mitochondria (OCA7 colocalization with AKAP1). For comparison, the *horizontal line* in both graphs represents the mean Pearson correlation coefficient for OCA7-EGFP colocalization with AKAP1-FRB-BFP2 for cells expressing WT Rab32. EGFP, enhanced GFP; ns, not significant; OCA7, oculocutaneous albinism type 7.

We utilized the FRB/FKBP12 heterodimerization system to induce mislocalization of Rab32 or Rab38 and test for OCA7 recruitment (Fig. 4*A*). We generated plasmids encoding AKAP1-BFP2-FRB, a mitochondria outer membrane marker with a fluorescent protein and heterodimerization domain, and mCherry-Rab32-CC/AA-FKBP12 containing a complementary heterodimerization domain. The Rab32 C-terminal cysteines were mutated to alanine to prevent geranyl-geranylation and insertion into melanosome membranes. In MNT1 cells expressing AKAP1-BFP2-FRB, mCherry-Rab32-CC/AA-FKBP12 and OCA7-EGFP cultured in the absence of the heterodimerizer (rapalog), mCherry-Rab32-CC/AA-FKBP12 exhibited cytosolic distribution as expected. Upon addition of rapalog, mCherry-Rab32-CC/AA-FKBP12 and OCA7-EGFP were recruited to mitochondria, indicating interaction with Rab32 recruited OCA7 to mitochondria (Fig. 4, *B* and *C*). Demonstrating specificity, while the constitutively active Rab32 mutant (Q85L) showed robust recruitment of OCA7-EGFP to mitochondria, the corresponding dominant negative Rab32 mutant (T39N) displayed reduced recruitment of OCA7-EGFP. Consistently, the constitutively active S2 mutant form or Rab32 (Q85L, Y95A) showed reduced recruitment of OCA7-EGFP to mitochondria (Fig. 4, *B* and *C*).

Cells showed varying degrees of AKAP1-BFP2-FRB and mCherry-Rab32-CC/AA-FKBP12 heterodimerization after addition of rapalog. Using regression analysis, we found cells having a higher degree of colocalization between AKAP1-BFP2-FRB and mCherry-Rab32-CC/AA-FKBP12 also had higher colocalization between AKAP1-BFP2-FRB and OCA7-EGFP, indicating a positive correlation between dimerization efficiency and OCA7-EGFP recruitment to mitochondria (Fig. 4*D*, *upper panel*). In contrast, cells having a high degree of colocalization between AKAP1-BFP2-FRB and dominant negative Rab32 (T39N) did not correlate with higher colocalization between AKAP1-BFP2-FRB and OCA7-EGFP (Fig. 4*D*, *lower panel*). Similar to Rab32, mislocalization to mitochondria of WT and constitutively active (Q69L) mCherry-Rab38-CC/AA-FKBP12 was sufficient to mislocalize OCA7-EGFP to mitochondria, whereas dominant negative (T23N) and S2 (Y79A) mutants had reduced recruitment capacity (Fig. S3). Together, these results indicate that Rab32 and Rab38 have the ability to recruit OCA7 to membranes.

### The OCA7 C terminus is dispensable for recruitment to melanosomes

**Figure 5:**
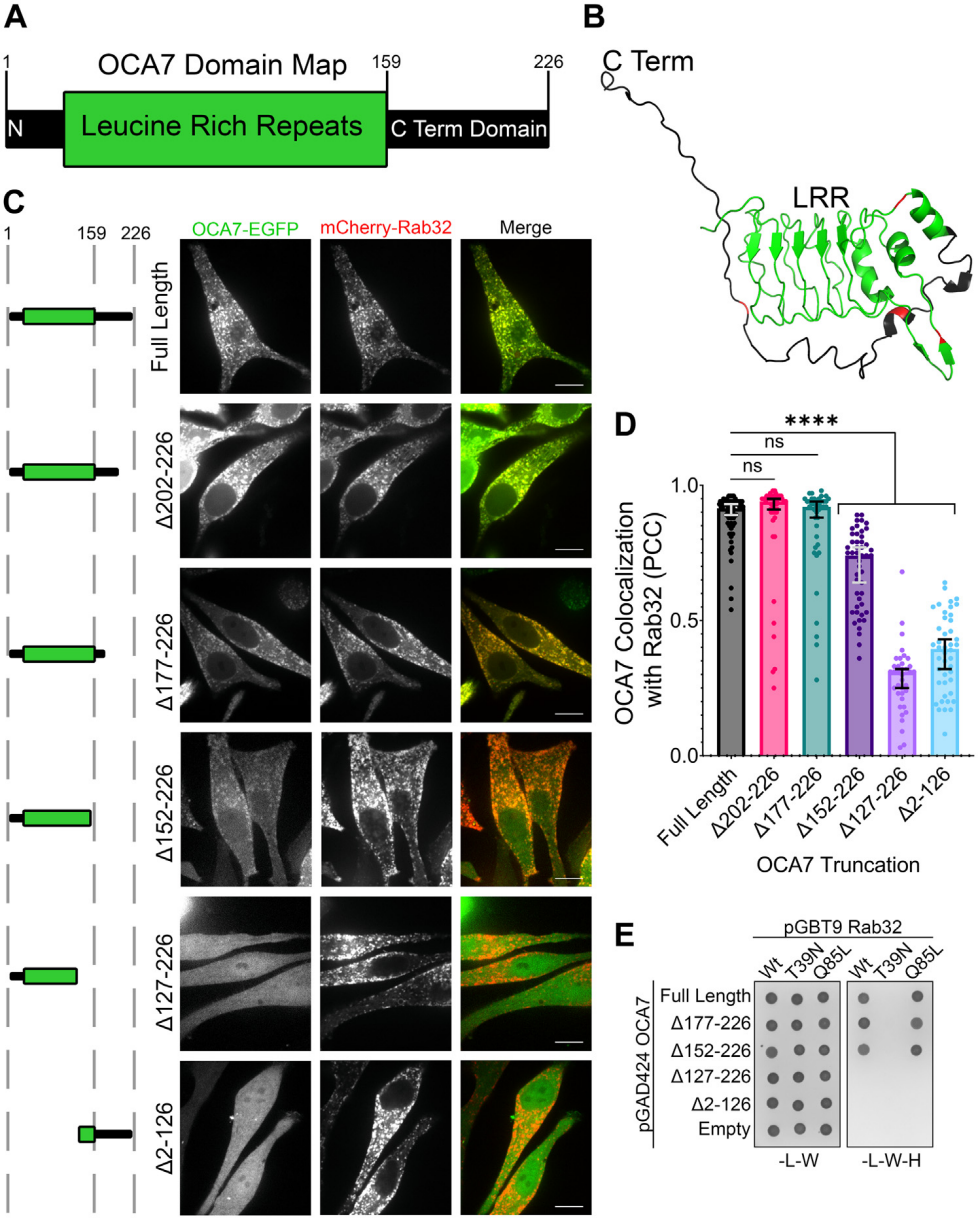
**The C terminus of OCA7 is dispensable for interaction with Rab32 and OCA7 recruitment to melanosomes.***A*, domain map depicting OCA7 with a leucine-rich repeat (LRR) domain and a disordered domain at the C terminus. Positions 1, 159, and 226 are marked to show position of residues relative to the LRR domain. *B*, predicted molecular structure of OCA7 generated using the Phyre2 protein fold prediction server showing LRR domain (*green*) and disordered C-terminal domain (*black*) (69). Residues highlighted in *red* indicate locations of each successive 25 amino acid truncation made for the experiment. *C*, live cell spinning disc confocal fluorescence microscopy images of MNT1 cells expressing full-length or truncated OCA7-EGFP with mCherry-Rab32. Truncation of the disordered C terminus had little or no effect on OCA7-EGFP colocalization with Rab32, but truncation of or into the LRR domain disrupted OCA7 punctate distribution. The scale bars indicate 10 μm. *D*, graph of PCC corresponding to images in (*C*). The graph shows median with error bars representing the 95% confidence interval. Colocalization was measured for n = 66, n = 48, n = 41, n = 48, n = 37, and n = 44 cells for OCA7 full length, Δ202 to 226, Δ177 to 226, Δ152 to 226, Δ127 to 226, and Δ2-126, respectively. The Kruskal–Wallis test was used to test significance. For statistical comparisons, ns and ∗∗∗∗ correspond to *p* > 0.05 and *p* < 0.0001, respectively. *E*, Y2H assay showing C-terminally truncated OCA7 maintains interaction with Rab32, but truncation of the LRR domain abolishes interaction. Growth on +histidine plates indicates successful cotransformation with pGBT9 and pGAD424 vectors, and growth on −histidine plates indicates interaction between the bait and prey proteins. The T39N mutation prevents Rab32 from exchanging GDP for GTP, keeping it in the “off” conformation. The Q85L mutation prevents Rab32 from hydrolyzing GTP, keeping it in the “on” conformation. OCA7, oculocutaneous albinism type 7; Y2H, yeast two hybrid.

Sequence analysis suggests that OCA7 consists of an N-terminal leucine-rich repeat (LRR) domain and a disordered C-terminal domain (Fig. 5, *A* and *B*). Colocalization analysis of full length or truncations of OCA7-EGFP revealed the C-terminal domain is dispensable for recruitment to melanosomes (Fig. 5, *A*–*D*). Larger C-terminal truncation including part of the LRR domain or deletion of LRR *via* N-terminal truncation drastically reduced OCA7-EGFP localization to melanosomes, rendering it cytosolic (Fig. 5, *A*–*D*). Y2H assays revealed that the C-terminal domain of OCA7 is also dispensable for interaction with Rab32 (Fig. 5*E*). Together, these results suggest that the OCA7 LRR domain is necessary and sufficient for interaction with Rab32 and recruitment to melanosomes.

### OCA7-KO MNT1 cells are hypopigmented

**Figure 6:**
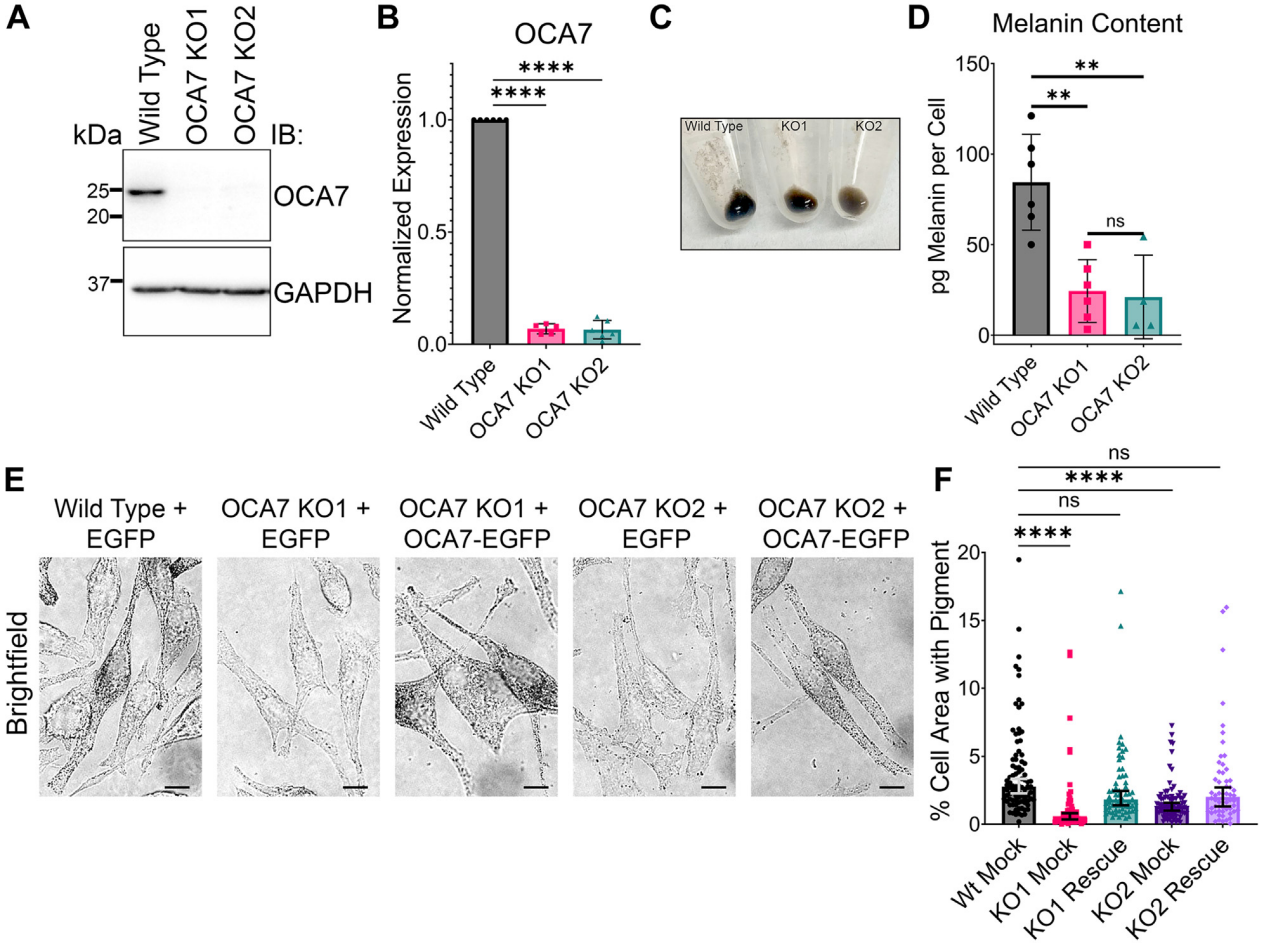
**OCA7-KO MNT1 cells are hypopigmented.***A*, IB analysis of total cell extracts from WT or OCA7-KO MNT1 cells showing OCA7 is depleted in both KO clones used in the study. *B*, quantification of OCA7 protein levels from IBs shown in (*A*) represented as mean ± SD normalized to WT (n = 6; OCA7-KO1, n = 5; OCA7-KO2, n = 6). *C*,image of one million WT or OCA7-KO cell pellets. *D*, quantification of melanin content in WT and OCA7-KO cells determined by bulk melanin quantification assay. Several independent experiments were quantified for WT (n = 6), OCA7-KO1 (n = 6), and OCA7-KO2 (n = 4) and represented as mean ± SD. *E*, brightfield images of fixed WT or OCA7-KO MNT1 cells transfected with EGFP (Mock) or OCA7-EGFP (rescue) plasmids. The scale bars indicate 10 μm. *F*, quantification of pigment in brightfield images depicted in (*E*) as the percent of the cell area meeting a *gray* value threshold, corresponding to the percent of the cell containing visible melanin. EGFP, enhanced GFP; IB, immunoblot; OCA7, oculocutaneous albinism type 7.

We employed CRISPR-Cas9 to knock out OCA7 in MNT1 cells to recapitulate OCA7 disease at the cellular level. Genomic DNA sequencing showed the gene was mutated in two clones (KO1 and KO2) (Fig. S4*A*). IB analysis confirmed the absence of the OCA7 protein in corresponding cell lysates (Figs. 6, *A* and *B* and S4*B*). Immunoprecipitation (IP) of endogenous OCA7 from MNT1 lysates with an anti-OCA7 antibody and IB with a different anti-OCA7 antibody resulted in strong detection of OCA7 in WT cells and no detection in OCA7-KO cells, confirming the KO cells do not express OCA7 and the ability of the antibodies to react against OCA7 (Fig. S4*B*). Anti-OCA7 antibodies also recognized recombinant OCA7 (Fig. S4*C*). Visual inspection of OCA7-KO MNT1 cell pellets containing one million cells revealed a reduction of pigmentation relative to WT cells (Fig. 6*C*). Melanin quantification of cell homogenates using a spectrophotometric assay confirmed melanin reduction in OCA7-KO cells (Fig. 6*D*). In addition, WT and OCA7-KO MNT1 cells were transfected with plasmids encoding EGFP (mock) or OCA7-EGFP (rescue) and imaged by brightfield microscopy confirming hypopigmentation in OCA7-KO cells, and that transient OCA7-EGFP expression rescued pigmentation levels (Fig. 6, *E* and *F*). These data validate the specificity of the OCA7-KO cells and that OCA7-EGFP is functional in pigmentation. Importantly, our findings are consistent with patients diagnosed with OCA7, who have light complexion but retain some skin pigment and tend to have blonde or light brown hair (5, 15, 34).

### PMEL is aberrantly processed in OCA7-KO MNT1 cells

**Figure 7:**
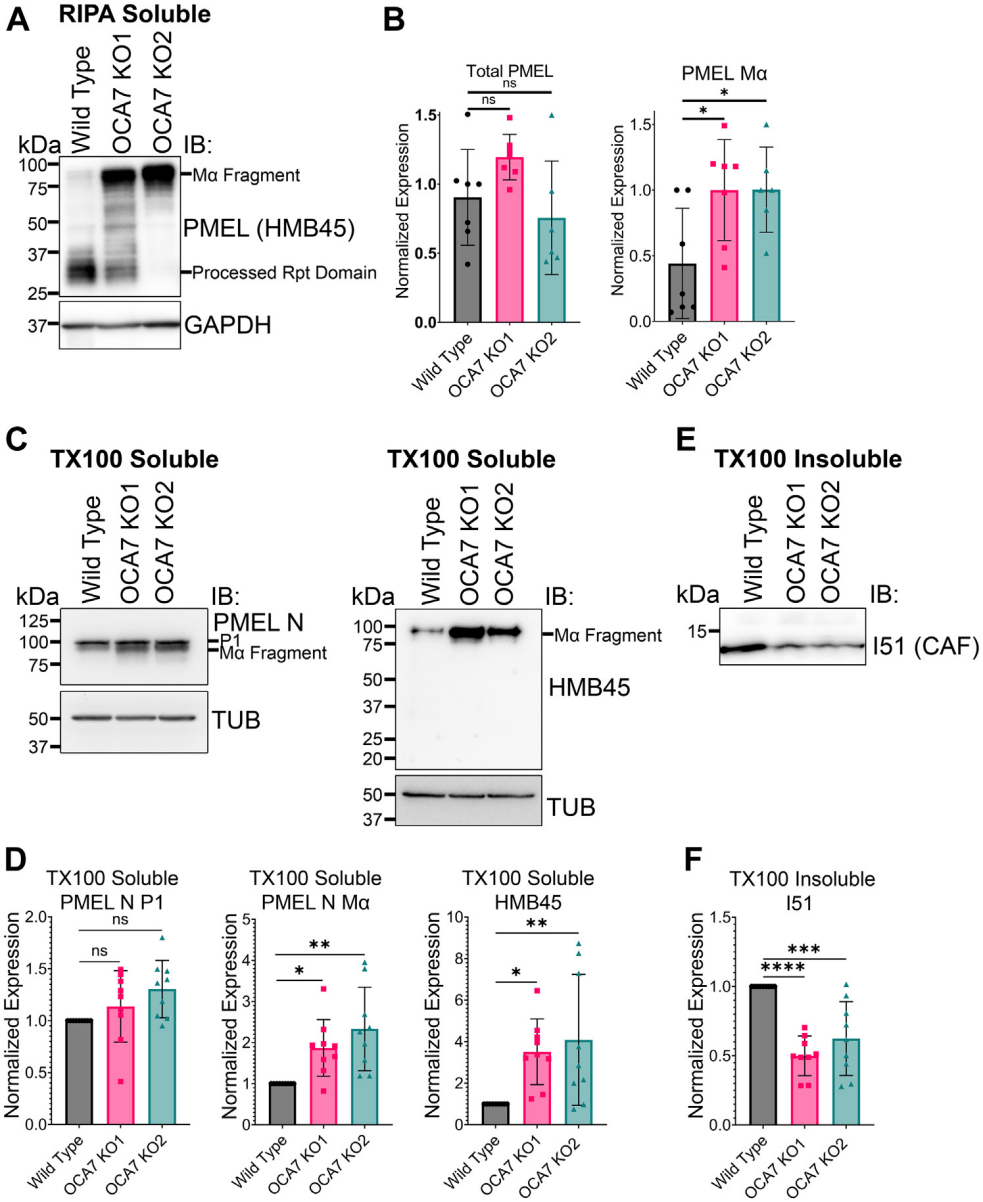
**PMEL is aberrantly processed in OCA7-KO MNT1 cells.***A*, IB analysis of RIPA buffer lysates using antibodies to PMEL (HMB45) show an accumulation of the Mα fragment in OCA7-KO cells. *B*, quantification of IBs depicted in (*A*) showing total HMB45 signal or Mα fragment only (mean ± SD; WT, n = 7; OCA7-KO1, n = 7; OCA7-KO2, n = 6). *C*, IB of cell lysates prepared with a buffer containing 1% TX100 from WT or OCA7-KO MNT1 cells probed with HMB45, PMEL N, or antitubulin antibodies. *D*, quantification of PMEL full length (P1) and Mα fragment from IBs shown in (*C*). The graphs show mean ± SD, n = 9 independent experiments were quantified. One-way ANOVA and post hoc Tukey tests were used to test for significance. ns, ∗, and ∗∗ correspond to *p* > 0.05, *p* < 0.05, and *p* < 0.01, respectively. *E*, IB of TX100 insoluble cell lysates from WT or OCA7-KO MNT1 cells probed with I51 antibody reactive toward the core amyloid fragment (CAF) of PMEL. *F*, quantification for IB shown in (*E*). The graph shows mean ± SD for n = 9 independent experiments. IBs for TX100 insoluble PMEL were normalized to TX100 soluble tubulin from the corresponding samples. Statistical significance was tested using one-way ANOVA and Tukey post hoc tests. ∗∗∗ and ∗∗∗∗ correspond to *p* < 0.001 and *p* < 0.0001, respectively. IB, immunoblot; OCA7, oculocutaneous albinism type 7; PMEL, premelanosome protein; RIPA, radioimmunoprecipitation assay; TX100, Triton X-100.

We sought to understand how pigment is reduced in OCA7-KO cells and reasoned that OCA7 might regulate total levels of the enzymes that make melanin or other proteins involved in melanosome biogenesis. IB analysis of total cell extracts generated from WT and OCA7-KO cells with radioimmunoprecipitation assay (RIPA) buffer failed to detect differences in the levels of the melanosome enzymes that synthesize melanin, tyrosinase, tyrosinase-P1, and tyrosinase-P2 (Fig. S5). LAMP2, Rab32, and Rab38 also showed unaltered steady-state levels in OCA7-KO cells (Fig. S5). PMEL protein is proteolytically processed in melanosomes and forms amyloid fibrils that function as a scaffold for melanin deposition and packing. The HMB45 antibody detects partially processed PMEL Mα fragment and further processed Rpt (repeat) domain (35, 36). Strikingly, HMB45 IB analysis of total cell extracts generated from OCA7-KO cells with RIPA buffer revealed an accumulation of the partially processed Mα fragment compared with WT cells (Fig. 7, *A* and *B*). Full-length PMEL and partially processed intermediates are soluble in Triton X-100 (TX100) buffers, whereas the highly proteolytically processed PMEL fragments present in fibrils are not, requiring high SDS concentration and high temperature for solubilization. To further test for a PMEL defect, we performed IB analysis with TX100 soluble and TX100 insoluble extracts from WT and OCA7-KO MNT1 cells. Both the PMEL N and HMB45 antibodies showed that OCA7-KO cells have an accumulation of TX100-soluble Mα fragment (Fig. 7, *C* and *D*). The PMEL N antibody also recognizes full-length PMEL (P1), which displayed normal steady-state levels in OCA7-KO cells (Fig. 7, *C* and *D*). The I51 antibody recognizes the so called “core amyloid fragment” (CAF) of insoluble PMEL fibrils integral to PMEL fibril structure (36, 37, 38, 39). IB analysis of TX100 insoluble fractions with I51 found less CAF in OCA7-KO cells compared with WT (Fig. 7, *E* and *F*). These findings suggest that PMEL processing is defective in steps after generation of the Mα fragment in OCA7-KO cells.

### OCA7-KO MNT1 cells have aberrant melanosomes and fewer striated melanosomes

**Figure 8:**
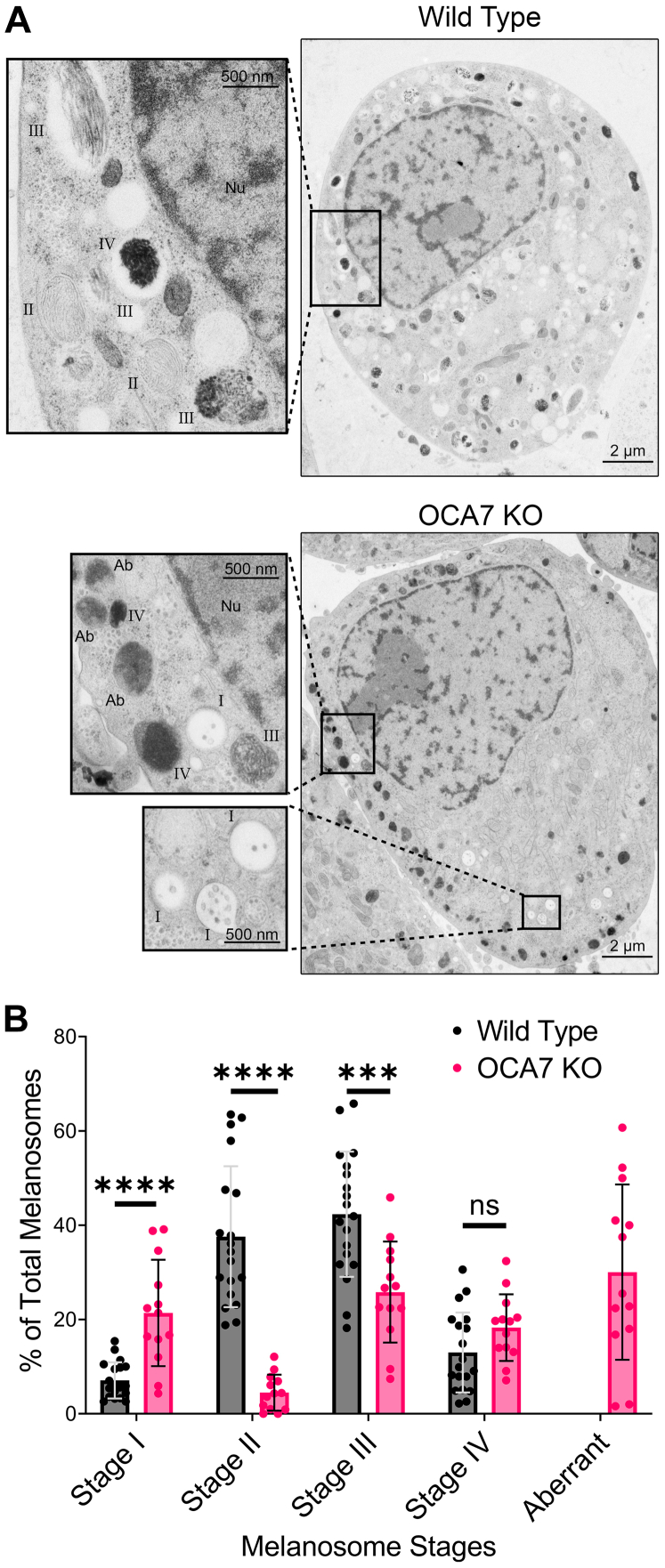
**OCA7-KO MNT1 cells have aberrant melanosomes and fewer striated melanosomes.***A*, thin section transmission electron micrographs of WT or OCA7-KO MNT1 cells (6800×). *Magnified insets* highlight examples of stage I, II, III, IV, or aberrant (Ab) melanosomes (30,000×). *B*, quantification of the percent of melanosomes in each stage per cell in WT and OCA7-KO cells depicted in images from (*A*) representing the mean ± SD (WT, n = 19 cells; OCA7-KO1, n = 13 cells). OCA7, oculocutaneous albinism type 7.

PMEL fragments form electron dense amyloid fibril striations inside melanosomes that get packed with melanin pigment (18). Given a PMEL processing defect was detectable by IB, we hypothesized a defect in PMEL fibril striation, and melanin packing would occur in OCA7-KO cells and be visible by electron microscopy. WT and OCA7-KO MNT1 cells were high pressure frozen and analyzed by thin section transmission electron microscopy. As expected, WT cells had few stage I melanosomes, an abundance of stage II and stage III melanosomes having clear PMEL fibrils, and several stage IV melanosomes having PMEL fibrils packed with melanin (Fig. 8). In contrast, quantification showed that OCA7-KO cells had significantly more stage I and less stage II and stage III melanosomes (Fig. 8). In addition, OCA7-KO cells had numerous aberrant melanosomes containing a disordered and less electron dense cloud of melanin but lacking stereotypical striations. These results are consistent with a defect in PMEL fibrillation and melanosome biogenesis in OCA7-KO cells.

### Melanosomes are more acidic in OCA7-KO MNT1 cells

**Figure 9:**
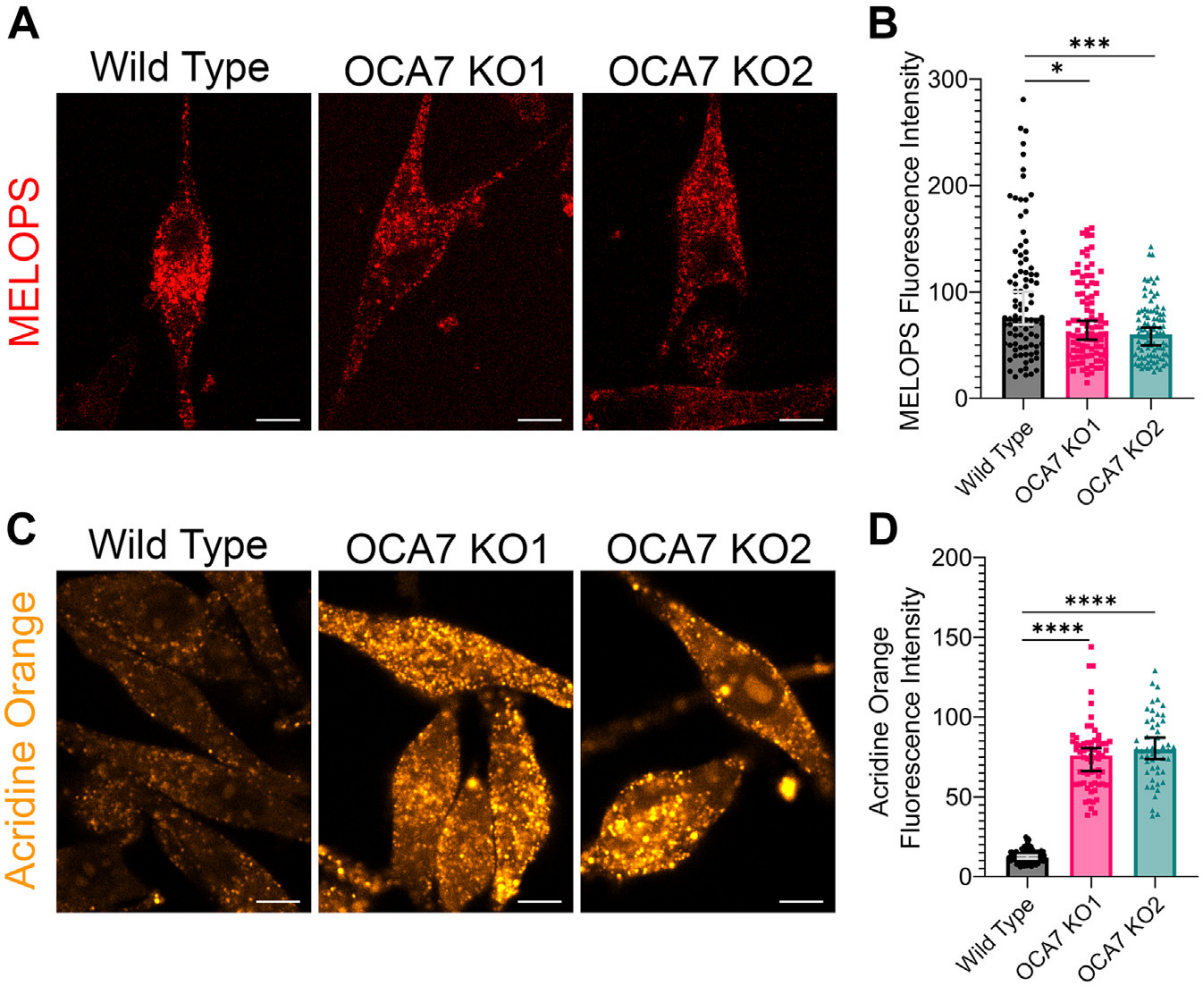
**OCA7-KO MNT1 cells have melanosomes with lower luminal pH.***A*, laser scanning live cell microscopy images of WT and OCA7-KO MNT1 cells expressing MELOPS. The scale bars indicate 10 μm. *B*, quantification of images from (*A*). The *graph* shows the median MELOPS fluorescence intensity with error bars representing the 95% confidence interval. n = 90, n = 97, and n = 94 cells were analyzed for WT, OCA7-KO1, and OCA7-KO2, respectively. Significance was tested using the Kruskal–Wallis test. ∗ and ∗∗∗∗ correspond to *p* < 0.05 and *p* < 0.0001, respectively. *C*, laser scanning live cell confocal microscopy images of WT and OCA7-KO MNT1 cells stained with acridine orange. The scale bars indicate 10 μm. *D*, quantification of images from (*C*). The graph shows the median fluorescence intensity with error bars representing the 95% confidence interval. n = 121, n = 65, and n = 50 cells were analyzed for WT, OCA7-KO1, and OCA7-KO2, respectively. Significance was tested using the Kruskal–Wallis test. ∗∗∗∗ corresponds to *p* < 0.0001. MELOPS, MElanosome LOcalized pH Sensor; OCA7, oculocutaneous albinism type 7.

The aberrant processing of PMEL likely contributes partially, but not entirely, to the hypopigmentation observed in OCA7-KO MNT1 cells. We reasoned OCA7-KO cells may have a broader defect in melanosome maturation. To test for a melanosome pH defect in OCA7-KO MNT1 cells, we used a MElanosome LOcalized pH Sensor (MELOPS) we previously developed. MELOPS is based on the OCA2 protein and contains a pH-sensitive fluorophore exposed to the melanosome lumen. Live cell imaging of OCA7-KO MNT1 cells expressing MELOPS showed a small but significant reduction in sensor fluorescence relative to WT cells, which corresponds to a more acidic pH (Fig. 9, *A* and *B*). The MELOPs range of detection is more suitable to sense pH increases than decreases (23). It is possible that the subtle fluorescence intensity decrease detected in OCA7-KO cells may represent an underestimation of the phenotype. In addition, because of the natural localization of OCA2, MELOPs is more likely to report on more mature melanosomes, whereas OCA7 is enriched in less mature melanosomes (23). To corroborate these results, we incubated WT and OCA7-KO MNT1 cells with acridine orange, a dye that accumulates in organelles with low pH including melanosomes and endolysosomal organelles (40). Live cell imaging analysis showed significantly brighter fluorescence in OCA7-KO cells compared with WT cells, confirming they have more acidic organelles (Fig. 9, *C* and *D*). Abnormal luminal pH is known to negatively influence activity of melanogenic enzymes and likely explains why OCA7-KO cells are hypopigmented (41).

### OCA7 functions independently of TPC2

We initially discovered OCA7 at melanosomes through proximity biotinylation by the melanosome-localized ion channel TPC2-BioID2-hemagglutinin (HA). In the present study, we found that OCA7 deficiency reduces pigment and pH of melanosomes in MNT1 cells. In a previous study, we established that TPC2 depletion increases pigment and pH of melanosomes in MNT1 cells (23). Therefore, an enticing possibility is that OCA7 might work as a negative regulator of TPC2 to control pH and pigment of melanosomes. We reasoned if OCA7 primary function is to regulate TPC2, then depletion of OCA7 in a TPC2-KO background should not influence pigmentation or pH. Starting with TPC2-KO MNT1 cells used in previous studies, we knocked out OCA7 resulting in TPC2/OCA7 double-KO cells. IBs confirmed efficient depletion of OCA7 in the bulk population of double-KO cells (Fig. S6*A*). Visualization of cell pellets indicated an obvious reduction in pigmentation in the TPC2/OCA7 double-KO cells compared with parental TPC2-KO cells, suggesting OCA7 does not control pigmentation through TPC2 (Fig. S6*B*). Furthermore, TPC2/OCA7 double-KO cells had heightened acridine orange accumulation relative to TPC2-KO cells, suggesting OCA7 regulates melanosome lumen pH independent of TPC2 (Fig. S6, *C* and *D*). In addition to regulating pH of melanosomes, TPC2 was previously found to regulate basal cytosolic calcium concentration in the vicinity of melanosomes, presumably *via* calcium release from the melanosome lumen. Here, we tested if OCA7 regulates this TPC2 activity (23). TPC2-GCaMP6-mCherry was expressed in WT and OCA7-KO MNT1 cells such that the calcium sensor GCamP6 was localized on the cytosolic side of the melanosome membrane. Determination of the GCaMP6/mCherry fluorescence intensity ratio showed no difference between WT and OCA7-KO cells suggesting that calcium regulation by TPC2 is independent of OCA7 (Fig. S6, *E* and *F*). Finally, we tested if OCA7 and TPC2 interact physically using co-IP. IB confirmed efficient IP of endogenous OCA7 but failed to detect pulldown of overexpressed TPC2-BioID2-HA (Fig. S6*G*). Together, we conclude OCA7 and TPC2 probably do not work with each other functionally or physically despite their close proximity at melanosomes.

## Discussion

OCA7 was previously hypothesized to regulate melanocyte differentiation. This idea stemmed from a study finding that depletion of an OCA7 homolog in sea squirt led to embryonic developmental defects resembling β-catenin depletion (42). Another study found that OCA7 knockdown appeared to reduce the number of melanocytes in zebrafish as assessed by visualization of pigment at the organismal level (15). However, the same study also found that OCA7 mRNA expression occurs downstream of MITF, a transcription factor controlling expression of many melanocyte-specific proteins. Therefore, it is unclear if OCA7 is involved in the upstream process of melanocyte differentiation. OCA7 patients retain the capacity to synthesize melanin, suggesting melanocytes are still present, and the idea that OCA7 is involved in melanocyte differentiation has not been supported in humans or mammals. Here, we provide evidence for an alternative model in which OCA7 functions at melanosomes where it is needed for normal organelle biogenesis and function (Fig. 10).

First, several lines of evidence show that OCA7 is a melanosome-associated peripheral membrane protein enriched in maturing melanosomes: (i) using an unbiased BioID2 screen, we discovered OCA7 in the proximity of TPC2, a melanosome membrane protein. (ii) Fluorescence microscopy with an array of organelle markers corroborated that OCA7 localizes to melanosomes, especially the less mature forms of these organelles. (iii) FRAP and biochemical approaches showed OCA7 is present both in the cytosol and melanosome membranes quickly exchanging between the two pools. (iv) Y2H experiments showed OCA7 interacts with Rab32 and Rab38, two proteins known to function in melanosome biogenesis. This interaction occurs *via* a Rab canonical effector–binding surface and the OCA7 LRR domain and likely mediates OCA7 recruitment to melanosomes.

Second, results indicate OCA7 functions in the biogenesis of melanosomes. OCA7-KO MNT1 cells are hypopigmented but retain expression of melanocyte-specific markers tyrosinase, tyrosinase-p1, and tyrosinase-p2, consistent with the idea that OCA7 regulates pigmentation independent of melanocyte differentiation. Importantly, OCA7-KO MNT1 cells transiently transfected with OCA7-EGFP quickly recover normal pigmentation, thus providing a validated system to study OCA7 at the cellular level. With this cell model, we found OCA7 functions at early stages of melanosome biogenesis, specifically in the transition from stage I to stage II. This is demonstrated by the increased numbers of stage I but reduced numbers of stage II and stage III melanosomes in OCA7-KO MNT1 cells determined by electron microscopy. Lack of striations typical of stage II and stage III melanosomes is consistent with the PMEL processing defect postgeneration of the Mα fragment resulting in the accumulation of Mα and reduced CAF observed by IB. Abnormal melanosome luminal pH in OCA7-KO cells may affect the activity of enzymes that cleave PMEL in stage I melanosomes, proprotein convertase and BACE2, further explaining the maturation defect (43, 44). Aberrant pigmented melanosomes observed by electron microscopy in OCA7-KO MNT1 cells likely reflect the inability of melanin to deposit and concentrate because of reduced PMEL striations. Consistently, similar melanosomes with heterogeneous melanin deposition were observed in PMEL-KO mice and BACE2-KO mice (44, 45). Together, the data show that OCA7 works in early stages of melanosome biogenesis.

OCA7-KO MNT1 cells have a defect in PMEL processing, but it is unclear if a PMEL defect alone would lead to OCA. PMEL-KO mice have only a very subtle pigmentation deficiency, and human PMEL variants are associated with pigmentary glaucoma but not OCA (45, 46). Thus, the PMEL defect alone may not fully explain the OCA7 pigmentation phenotype in patients and MNT1-KO cells. Melanosome lumen pH is known to affect the activity of tyrosinase, the key enzyme in melanin synthesis (40, 41). The lower melanosome luminal pH in OCA7-KO MNT1 cells detected by both MELOPS and acridine orange methods is consistent with the reduced melanin levels observed in these cells and OCA7 patients. Therefore, it is likely that overall OCA7 hypopigmentation is the result of both PMEL processing and fibrillation defect as well as decreased melanin synthesis because of reduced melanosome pH. Future work will investigate how OCA7 regulates melanosome pH. We were unable to confirm a functional or physical interaction between OCA7 and TPC2, but we hypothesize OCA7 may influence pH by working with other ion channels or proton pumps.

In accordance with our findings, it was previously shown that Rab32 deficiency leads to increased PMEL Mα levels (47). Our results suggest that OCA7 works with Rab32 to regulate PMEL fibrillation and melanosome biogenesis. It is worth noting that OCA7 and Rab32 are expressed in several cell types besides melanocytes suggesting OCA7 may also function with other lysosome-related organelles (proteinatlas.org/ENSG00000148655-LRMDA, Fig. S7*A*) (48). For example, Rab32 regulates the maturation of phagosomes in macrophages during pathogen clearance, resides on lysosomes in hepatocytes, and is involved in the biogenesis of platelet dense granules in megakaryocytes (49, 50, 51, 52, 53). Interestingly, a recent case report described two OCA7 patients who faced recurring respiratory infections in addition to the expected OCA manifestations suggesting OCA7 may in fact have broader functions (34). Adding to this possibility, recent genome-wide association studies listed SNPs in intronic regions of the OCA7 gene to be associated with coronavirus disease 2019 susceptibility in non-Europeans and with platelet aggregation, suggesting OCA7 may have functions in immune cells and megakaryocytes/platelets, respectively (54, 55). IB analysis of lysates made from imMKCL cells, a well-validated human megakaryocyte cell line, confirmed OCA7 protein expression (Fig. S7*B*) (56, 57, 58). Expression of OCA7-EGFP in differentiated imMKCL cells revealed its localization to membranes of vacuolar compartments that partially overlapped with the platelet dense granule marker VMAT2-mCherry (Fig. S7, *C* and *D*). These results lend credence to the idea that OCA7 may regulate the function of other lysosome-related organelles in addition to melanosomes. Such a scenario would suggest that the disease caused by OCA7 mutation may impact additional cell types and be better described as a syndrome, such as the Hermansky–Pudlak syndrome (59).

In summary, the present study revealed OCA7 is a melanosome protein that regulates organelle biogenesis and function. The data place OCA7 as one of the few factors known to mediate early stages of melanosome maturation. While important mechanistic clues emerge from our results, more work needs to be done to fully understand OCA7. For example, Rab32 and Rab38 have been implicated both in forward traffic to maturing melanosomes as well as retrieval from melanosomes to endosomes (26, 47, 60, 61). Future studies should explore if OCA7 function is linked to Rab32/Rab38 forward or retrieval traffic and better define how OCA7 regulates organelle pH. Finally, the possibility that OCA7 regulates other lysosome-related organelles in addition to melanosome deserves serious consideration both from basic cell biology and clinical standpoints.

## Experimental procedures

### Cell culture and transfection

MNT1 cells were cultured as previously described (26). For microscopy experiments, 0.3 × 10^6^ cells were resuspended in 20 μl Lonza SF solution, mixed with 0.4 μg of each plasmid, transferred to 16-well nucleocuvette strips, and nucleofected using program DS-137 in a Lonza 4D nucleofector. After 10 min, cells were transferred to 35 mm glass bottom dishes with 2 ml 37 °C KGM Gold media and grown 24 to 72 h. Nonidentified human neonatal primary epidermal melanocytes (Thermo Fisher Scientific; catalog no.: C0025C) were cultured in supplemented Medium 254 per manufacturer instructions and nucleofected as described for MNT1 cells. imMKCL cells were cultured as previously described (58, 62) and nucleofected as described for MNT1 cells except program EN-138 was used for nucleofection. About 24 h after transfection, imMKCL cells were transferred to Matrigel-coated glass bottom dishes, and the media were replaced with fresh media lacking doxycycline to induce differentiation. imMKCL cells were imaged after 72 h in differentiation media.

### Plasmids

Plasmids were generated using Infusion Cloning (Takara Bio, Inc). Primers were designed to have 15 base pair 5′ extensions to overlap with linearized vectors for infusion cloning. Primer pairs are listed in Table S1. OCA7, AP1M1, RabIF, and EXOSC5 were cloned from cDNA originating from MNT1 cells using CloneAmp HiFi PCR Premix (Takara Bio, Inc) and the Infusion cloning system (Takara Bio, Inc). To acquire cDNA, total RNA was prepared from MNT1 cells using the RNEasy kit (Qiagen) and converted to cDNA using iScript Reverse Transcriptase Supermix (Bio-Rad). To generate pEGFP-N3-OCA7, OCA7 was amplified from MNT1 cDNA and inserted by infusion into a linear pEGFP-N3 vector linearized by restriction digestion with HindIII-HF (NEB) and ApaI (NEB). To generate pET30-OCA7-6His for recombinant expression, OCA7 was amplified from pEGFP-N3-OCA7 and inserted by infusion cloning into pET-30a(+) that was linearized with NdeI and NotI-HF. To generate pGAD424-OCA7 for Y2H analysis, OCA7 was amplified from pEGFP-N3-OCA7 and inserted by infusion cloning into pGAD424 linearized with EcoRI-HF and SalI-HF. To generate truncations in pGAD424-OCA7 and pEGFP-N3-OCA7 plasmids, inverse PCR was performed with primers designed to exclude unwanted sequence and add complementary 15 base pair 5′ overhangs. After PCR, the linear plasmids were closed by infusion cloning. pGBT9-AP1M1, pGBT9-RabIF, and pGBT9-EXOSC5 were generated by amplifying each ORF from MNT1 cDNA and inserting by infusion cloning into pGBT9 linearized by EcoRI-HF and SalI-HF. AKAP1-FRB-BFP2 was generated by digesting AKAP1(31)-FRB-iRFP (gifted by Gerry Hammond, Addgene #139315 (63)) to remove iRFP and using infusion cloning to insert mBFP2 amplified from pmBFP2-KDEL. To generate pmCherry-Rab32-CC/AA-FKBP and pmCherry-Rab38-CC/AA-FKBP, pmCherry-C2-Rab32 or pmCherry-C2-Rab38 were linearized and mutagenized by inverse PCR to mutate cysteines to alanines. Then FKBP12 was amplified from FKBP12-TOPO-M13-pUC (generously gifted by Soham Chanda) and inserted into the linearized vectors by infusion cloning. pmCherry-C2-CD63 was subcloned from pEGFP-C2-CD63 (gifted from Paul Luzio, Addgene #62964) by restriction digestion with HindIII-HF and BamHI-HF and T4 Ligation (NEB) into pmCherry-C2 digested with the same enzymes. To generate TPC2-GCaMP6-mCherry, TPC2-GCaMP6 (Addgene #80147) was first mutated to remove the stop codon and add a C-terminal AgeI restriction site. Then, pmCherry-N3 was digested with Xma1 and Not1, and the mCherry fragment was ligated into TPC2-GCaMP6 digested with AgeI and Not1, generating TPC2-GCaMP6-mCherry. To generate TPC2-BioID2-HA, TPC2 was amplified from pEGFP-N3-TPC2 (Addgene #80153) and inserted into MCS-Linker-BioID2-HA (gifted from Kyle Roux, Addgene #74224) linearized by inverse PCR. Plasmids were confirmed by Sanger sequencing.

pGBT9-Rab32, pGBT9-Rab38, pGBT9-Rab8, pGBT9-Rab11a, pGBT9-Rab10a, pGBT9-Rab27a, and their mutants for Y2H were generated and validated for a previous study (27). pmCherry-C2-Rab32, pmCherry-C2-Rab38, pmCherry-N3-tyrosinase, pmCherry-N3-tyrosinase-p1, pmCherry-C2-Rab27a, pmCherry-C2-Rab7a, pmCherry-C2-Rab11a, pmCherry-C2-Rab5a, pmRFP-N1-SKL, pmBFP2-KDEL, piRFP-N1-TPC2, MELOPS, and VMAT2-mCherry for microscopy experiments were all generated and validated for previous studies (23, 26, 27, 52, 62, 64). pmaxGFP is from Lonza.

### Antibodies

Primary antibodies used were C10orf11/LRMDA/OCA7 (catalog no.: PA5-61519; Thermo Fisher Scientific, 1:1000 dilution), C10orf11/LRMDA/OCA7 (catalog no.: HPA050419; Atlas Antibodies, 1:1000 dilution), tubulin (catalog no.: T9026; Sigma, 1:100,000 dilution), GAPDH (catalog no.: 60004-1-Ig; ProteinTech, 1:500,000 dilution), PMEL N (E-7; catalog no.: sc-377325, 1:1000 dilution), PMEL Rpt (catalog no.: HMB45; Dako, 1:1000 dilution), PMEL CAF (I51; generously provided by Michael Marks and Ralf Leonhardt, 1:1000 dilution), CD63 (catalog no.: sc-5275; 1:1000 dilution), CD81 (B-11; catalog no.: sc-166029, 1:2000 dilution), tyrosinase (T311; catalog no.: sc-20035, 1:1000 dilution), tyrosinase-P1 (MelV, TA99; catalog no.: sc-58438, 1:1000 dilution), tyrosinase-P2 (C-9; catalog no.: sc-74439, 1:1000 dilution), LAMP2 (H4B4; catalog no.: sc-18822, 1:1000 dilution), and HA tag-horseradish peroxidase (Cell Signaling; catalog no.: 2999S, 1:1000 dilution). Rab32 and Rab38 are noncommercial rabbit polyclonal antibodies previously characterized and used at 1:1000 dilution (26). Secondary antibodies were horseradish peroxidase-conjugated antimouse immunoglobulin G (IgG) (catalog no.: NA931; Cytiva, 1:7500 dilution) and anti-rabbit IgG (catalog no.: NA934; Cytiva, 1:7500 dilution) for IB.

### CRISPR–Cas9–mediated KO

*OCA7/LRMDA* KO in MNT1 cells was achieved by nucleofecting Cas9/CRISPR RNA (crRNA)/transactivating CRISPR RNA (tracrRNA) ribonucleoprotein complexes into WT MNT1 cells according to the manufacturer’s protocol (IDT Alt-R CRISPR–Cas9 System). Two predesigned crRNAs, Hs.Cas9.LRMDA.1.AC and Hs.Cas9.LRMDA.1.AE, were selected from IDT to target *OCA7/LRMDA* exon 2 with high specificity (Table S2). To generate each ribonucleoprotein complex, 5 μl 200 μM crRNA and 5 μl 200 μM tracrRNA were mixed and heated at 95 °C for 5 min and allowed to cool to room temperature. About 2.1 μl PBS, 1.2 μl crRNA–tracrRNA duplex, and 1.7 μl 61 μM Cas9 were mixed and incubated at room temperature for 20 min. MNT1 cells (300,000 cells) were resuspended in 20 μl Lonza SF nucleofection solution and mixed with 5 μl of each ribonucleoprotein and 1 μl of IDT electroporation enhancer for a final volume of 31 μl. The mix was transferred to a well of a Lonza 16-well nucleocuvette strip and nucleofected using program DS-137. After nucleofection, 70 μl 37 °C media were added to the suspension, and the suspension was transferred to a 35 mm culture dish with 2 ml 37 °C MNT1 media and incubated for 72 h. For single-clone isolation, the OCA7-KO cells were trypsinized and diluted to five cells/ml in MNT1 media, and 200 μl was seeded into wells of 96-well plates. The following day, wells containing only a single cell were marked, and single cells were grown, replacing the media every 4 days. To genotype colonies, genomic DNA was prepared using the QuickExtract reagent according to the manufacturer’s instructions (Biosearch Technologies), and sequence surrounding exon 2 was amplified with primers listed in Table S3. PCR products were run in a 1.5% agarose gel in 1× Tris–acetate–EDTA buffer, and bands were excised and purified using a gel purification kit (GE Illustra). DNA was sequenced using Sanger sequencing, confirming both crRNAs had worked. OCA7 protein deficiency was confirmed by whole lysate IB and lack of signal after IP.

### Light microscopy

Spinning disk confocal fluorescence images were captured with an Olympus IX81 configured with an Andor iXon Ultra camera and phasor holographic photobleaching system (3i) using a 100×/1.40 numerical aperture (NA) oil immersion objective lens. The microscope was equipped with lasers for 405, 488, 561, and 640 nm and emission filter wheels for 521, 607, and 700 nm. Confocal Airyscan SR images were taken with a Zeiss LSM 900 with Airyscan 2 detector using a 63×/1.40 NA oil immersion or 40×/1.30 NA oil immersion objective lenses. Live cells were imaged in humidified stage incubators set to 37 °C with 5% CO_2_. Brightfield images for melanin pigment quantification were captured with a Keyence BZ-X710 wide-field fluorescence microscope using a 100×/1.45 NA objective lens. Images taken with the Olympus IX81 were analyzed with Slidebook6 software (3i). Colocalization analysis was performed by manually segmenting cells and creating corresponding masks for each cell, subtracting background fluorescence, and using the Colocalization module with Costes autothresholding to measure Pearson correlation coefficient. In some cases (Figs. 1, *B*, *C*, and 5*D*), a 2D Laplacian filter using a 3X3 kernel (-1,-1,-1;-1,8,-1;-1,-1,-1) was applied before colocalization analysis. ImageJ (NIH) was used to analyze fluorescence intensity in images taken with the Zeiss LSM 900. Cells were manually segmented in ImageJ, background was subtracted, and mean fluorescence intensity was measured. Line scan fluorescence intensity analysis was performed in ImageJ by making a straight line through regions of interest and plotting fluorescence intensity with the Plot Profile tool. Zeiss Zen Blue software was used for semiautomated cell segmentation and counting of single fluorescent melanosome puncta in melanocytes. To do so, binary masks were made using thresholding to segment individual cells, and an additional mask was made to highlight individual melanosome puncta. The Analyze Interactively module was used to check the automated cell segmentation and separate cells manually if necessary. Individual melanosomes were counted for each cell. Cell pigment was measured in brightfield images using ImageJ. Cells in images were manually segmented, and a threshold was set to highlight melanosomes inside cells. Then the area of the entire cell and the area filled by pigmented melanosomes were calculated, and the percent of the cell area with pigment was calculated.

### FRAP

For the FRAP experiment, 0.3 × 10^6^ WT MNT1 cells were nucleofected with 400 ng of pEGFP-N3-OCA7 and pmCherry-C2-Rab32 or pmCherry-C2-CD63 plasmids in 20 μl Lonza SF solution using Nucleofector 4D program DS-137, transferred to glass bottom microscopy dishes, and incubated for 72 h with KGM Gold media. On the day of imaging, the media were replaced with fresh media containing 10 μM nocodazole and incubated for approximately 90 min prior to imaging to prevent melanosome movement on microtubules. During imaging, 2 μm diameter circles were made in Slidebook6 to target melanosomes with the Phasor photobleaching device. For each cell, three separate melanosomes were photobleached, and their fluorescence intensity was recorded during time-lapse imaging. Time lapses acquired 10 frames at 1 s intervals before photobleaching, photobleaching ensued for 1 s, and an additional 190 frames were captured to measure recovery. The phasor was set to photobleach each area with the 488 nm laser and the 561 nm laser for 500 ms each. To analyze FRAP curves, fluorescence intensity of photobleached regions of interest (ROIs) was plotted using the Slidebook6 FRAP Analysis module and corrected for imaging induced photobleaching using an unbleached ROI as a reference. Photobleach-corrected FRAP curves were compiled in GraphPad Prism (GraphPad Software, Inc) and modeled using a one-phase decay curve fitting to calculate T1/2 of recovery for OCA7-EGFP.

### Rab32/38 mislocalization assay

For the induced dimerization experiments, 0.3 × 10^6^ WT MNT1 cells were nucleofected with 200 ng of pEGFP-N3-OCA7, 500 ng AKAP1-FRB-BFP2, and 400 ng pmCherry-C2-Rab32-CC/AA-FKBP or pmCherry-C2-Rab38-CC/AA-FKBP plasmids in 20 μl Lonza SF solution using Nucleofector 4D program DS-137, transferred to glass bottom microscopy dishes, and incubated for 24 h with KGM Gold media. Dishes were first imaged in the absence of dimerization reagent. Then, the media were replaced with fresh media containing 100 nM rapalog (Takara A/C heterodimerizer), and cells were incubated for 1 h to achieve efficient dimerization. After incubating with rapalog, dishes of cells were imaged again to test for OCA7-EGFP recruitment to Rab32/38-labeled mitochondria. Colocalization analysis was performed in Slidebook6.

### MELOPS melanosome pH experiment

For the MELOPS experiment, 0.3 × 10^6^ WT or OCA7-KO MNT1 cells were nucleofected with 450 ng MELOPS plasmid and 200 ng pmaxGFP soluble GFP plasmid (Lonza) as described in the Cell culture and transfection section. Cells were incubated in KGM Gold media for 24 h in 35 mm glass bottom dishes before imaging with a Zeiss LSM900 set to Airyscan SR mode with excitation set to 587 nm and detection set to 565 to 700 nm. During imaging, transfected cells were located with the green fluorescence channel (488 nm excitation and 490–575 nm detection) to prevent bias. MELOPS was quantified by measuring red fluorescence intensity of manually drawn ROIs in ImageJ.

### Acridine orange staining of acidic organelles

For acridine orange experiments, 0.3 × 10^6^ WT or OCA7-KO MNT1 cells were seeded in 35 mm glass bottom dishes with 2 ml 37 °C KGM Gold media and grown for 24 h. To stain cells, media were aspirated from the dish, and cells were incubated with 2 ml acridine orange (Bio-Rad, ICT937) diluted to 1 μM in KGM Gold media for 30 min in a humidified cell culture incubator at 37 °C and 5% CO_2_. After the incubation, cells were washed twice for 1 min with 2 ml 37 °C PBS. After washing, 2 ml fresh KGM Gold media were added to the dish, and the cells were placed in the humidified stage incubator of a Zeiss LSM900 microscope and immediately imaged in confocal mode with excitation set to 488 nm and detection of 400 to 700 nm. Images were analyzed by measuring fluorescence intensity of manually drawn ROIs in ImageJ.

### Electron microscopy

Pelleted cells were resuspended in growth media supplemented with cryoprotectant (2% sucrose and 150 mM d-mannitol) and spun down to a loose pellet. A few microliters of pelleted cells were subjected to high pressure frizzing using a Wohlwend Compact 02 high pressure freezer (Technotrade International) as previously described (65). Frozen samples were then freeze-substituted in anhydrous acetone containing 2% osmium tetroxide and 0.2% uranyl acetate and embedded in Epon/Araldite resin. Thin sections (80 nm) were cut using a Leica UCT ultramicrotome, collected on Formvar-coated copper slot grids and poststained with 2% aqueous uranyl acetate followed by Reynold’s lead citrate. The samples were imaged with a Tecnai T12 Spirit Transmission Electron Microscope, operating at 100 kV using an AMT charge-coupled device camera. Each cell analyzed was first imaged at low magnification (6800×) followed by sufficient high-magnification captures (18,500–49,000×) of different cell regions to cover the entire cell (5–10 high-magnification pictures per cell). For each cell, melanosomes were identified by their typical morphology using the low-magnification image and then confirmed with the corresponding high-magnification images. Quantification was performed by counting the number of stage I, stage II, stage III, stage IV, and aberrant melanosomes observed for each cell and reporting them as percent of total melanosomes per cell.

### IB

About 1.5 × 10^6^ cells were seeded in 10 cm dishes with 10 ml MNT1 media and incubated for 72 h in a humidified 37 °C incubator. To harvest cells, dishes were washed with 5 ml ice-cold PBS, scraped into suspension with 5 ml ice-cold PBS using cell lifters, and pelleted at 200*g* at 4 °C for 5 min. Cell pellets were resuspended in 250 μl RIPA buffer (150 mM NaCl, 1% w/v TX100, 0.1% w/v SDS, 1% w/v deoxycholate, 20 mM Tris–HCl [pH 8.0], and 1 mM EDTA) or 1% TX100 in PBS with 1× protease inhibitor cocktail (0.2 mM 4-benzenesulfonyl fluoride hydrochloride, 0.15 μM aprotinin, 1.5 μM E-64, 1 μM leupeptin, and 1.5 μM bestatin) and lysed on ice for 15 min, gently agitating every 5 min. Lysates were centrifuged at 16,000*g* at 4 °C for 15 min to pellet melanin and insoluble debris. About 10 μl aliquots of each lysate were taken and diluted 1:10 for total protein quantification using a Bradford Assay (Bio-Rad Protein Assay) for normalized gel loading. About 100 μl of remaining lysate was mixed with 100 μl 2× Laemmli sample buffer (which contains 8% SDS) and incubated for 5 min at 96 °C. For IBs, 30 μg of total protein was loaded into precast 4 to 20% gradient polyacrylamide Tris glycine gels (Invitrogen Novex WedgeWell). Protein was transferred to polyvinylidene difluoride membranes, and membranes were blocked in 5% (w/v) milk Tris-buffered saline with Tween-20 (TBST) at room temperature for 1 h. Primary antibodies were diluted in 5% (w/v) milk TBST and incubated with blots overnight at 4 °C. Membranes were washed three times in TBST for 5 min, incubated with secondary antibodies diluted in 5% (w/v) milk TBST for 1 h at room temperature, washed three times in TBST for 5 min, and developed with ECL Prime chemiluminescence reagent (Cytiva). Blots were quantified in ImageJ using the gel analyzer tool after background subtraction. Band intensities were normalized to GAPDH or tubulin-loading controls.

For PMEL blots, TX100 insoluble lysates were prepared by resuspending the insoluble pellet from 1% (w/v) TX100 lysates with 1 ml 1% (w/v) TX100 and incubating on ice 15 min to wash. Suspended insoluble material was pelleted at 16,000*g* at 4 °C for 15 min. After aspirating the wash, pellets were resuspended in 1× Laemmli sample buffer (which contains 4% SDS), incubated 5 min at 96 °C in a heat block, vortexed, incubated five more minutes at 96 °C in a heat block, spun for 10 s at 16,000*g*, and the supernatant was loaded into gels. IB band intensities for TX100 insoluble lysates were normalized to tubulin signal from the corresponding TX100 soluble lysate IB.

### M/C fractionation

MNT1 cells were grown for 3 days to 70 to 90% confluency in a 10 cm culture dish with MNT1 media. To harvest, the dish was washed with ice-cold PBS, and cells were scraped into suspension in 5 ml PBS using a cell lifter. Cells were centrifuged for 5 min at 200*g* at 4 °C. The pellet was resuspended in 400 μl M/C buffer containing 10 mm Hepes, pH 7.5, 250 mm sucrose, 1 mm DTT, 1 mm EGTA, 0.5 mm MgCl_2_, 1 mm GTPγS, and 1× protease inhibitor cocktail. Cells were homogenized by passing through a 25-gauge needle, passing up and down 15 times. The resulting homogenate was centrifuged 15 min at 1000*g* at 4 °C to pellet nuclei and debris. The supernatant containing cytoplasm was transferred to ultracentrifuge tubes and centrifuged for 30 min at 60,000 RPM in a TLA 100.3 rotor (Beckman Coulter) at 4 °C. The supernatant containing cytosolic proteins was transferred to a separate tube, and TX100 was added for 1% (w/v) total, and the pellet containing the membrane fraction was solubilized in an equivalent volume of 1% (w/v) TX100 containing M/C buffer on ice for 15 min. M/C fractions were analyzed by IB.

### IP

For IP for Fig. S4*B*, 2 μg of rabbit anti-OCA7 (PA5-61519) or irrelevant rabbit IgG was prebound to 40 μl Protein A Sepharose beads (GE Healthcare), incubating for 30 min at room temperature with 1 ml IP buffer containing 50 mM Hepes (pH 7.4), 100 mM KCL, 1 mM DTT, 1 mM EDTA, 1× protease inhibitor cocktail, and 1% (w/v) TX100. After binding, beads were centrifuged at 2000*g* for 10 s, and the supernatant was aspirated before storing on ice. WT or OCA7-KO cells were grown in 15 cm culture dishes with 30 ml MNT1 media to 80 to 90% confluency. Dishes were washed with 10 ml ice-cold PBS, and cells were scraped into suspension in 10 ml ice-cold PBS using cell lifters, pelleted, resuspended in 1 ml IP buffer, and lysed on ice for 15 min with gentle agitation every 3 min. Crude lysates were centrifuged at 16,000*g* at 4 °C for 15 min. Supernatants were incubated with 100 μl Protein A Sepharose for 10 min on ice to remove proteins that nonspecifically bind to the beads. Samples were then centrifuged at 2000*g* for 10 s to pellet the Sepharose. Next, 100 μl of the supernatant was taken as input, and the remaining supernatant was incubated with the antibody-bound Protein A Sepharose for 1 h at 4 °C, rotating end over end. After binding, samples were centrifuged at 2000*g* for 10 s, and the supernatant was aspirated. Beads were washed three times with 1 ml IP buffer containing 0.1% (w/v) TX100 and once with IP buffer lacking TX100, centrifuging at 2000*g* for 10 s. After the last wash, 40 μl Laemmli sample buffer was added to the beads, and samples were stored at −20 °C before IB for OCA7 and tubulin. For the co-IP experiment in Fig. S6*G*, a slightly modified protocol was used. About 3.0 × 10^6^ MNT1 cells were nucleofected with 4 μg TPC2-BioID2-HA plasmid and seeded into a 10 cm dish with 10 ml MNT1 media. Untransfected MNT1 cells were used as a control. Cells were harvested for co-IP at 72 h and lysed in 500 μl IP buffer consisting of PBS with 1% (w/v) TX100 and 1× protease inhibitor cocktail for 30 min on ice. Insoluble material was pelleted at 16,000*g* for 15 min at 4 °C, and the supernatant was incubated with 2 μg of rabbit anti-OCA7 antibody (PA5-61519) for 30 min and then incubated with 40 μl Protein A Sepharose bead slurry for 90 min. Next, the beads were washed three times with 500 μl of PBS with 0.1% TX100. Finally, OCA7 and associated protein was eluted with 60 μl 1× Laemmli sample buffer.

### Recombinant OCA7 expression

Recombinant OCA7 protein encoded in pET30(+)-OCA7-6his plasmid was expressed in BL21 codon plus *Escherichia coli* and purified using the TALON cobalt affinity resin (Thermo Scientific; catalog no.: 89964) as described previously (66).

### Y2H assay

Competent AH109 strain of yeast was prepared and frozen as described, and Y2H analysis was performed essentially as described (67, 68). To transform the AH109 yeast, cells were resuspended in 375 μl 40% (w/v) PEG 3350 with 0.1 M lithium acetate and mixed with 25 μl of carrier DNA (salmon sperm DNA previously boiled for 5 min and cooled on ice) for 0.125 mg/ml final concentration. About 500 ng of each pGBT9-Bait and pGAD424-Prey plasmid was mixed with the suspension, and the suspension was briefly vortexed and incubated at 30 °C for 30 min. Yeast suspensions were briefly vortexed and incubated for 15 min at 42 °C. After the incubations, yeast were centrifuged for 2 min at 1000*g* at 4 °C. Pellets were resuspended in 300 μl of water, and 100 μl was plated on synthetic dropout media lacking leucine and tryptophan (SD -L-W) media to select for yeast cells containing both plasmids, and plates were incubated for 72 h at 30 °C. Colonies were isolated and subcultured on new plates for 24 h at 30 °C. Liquid cultures were prepared by placing colonies into culture tubes containing 2 ml SD -L-W and grown overnight. Saturated cultures were diluted 1:10 in water, and 3 μl spots were made on SD -L-W, SD -L-W-H, SD -L-W-H + 3-amino-1,2,4-triazole plates. Plates were imaged after 72 h of incubation at 30 °C. Growth on plates lacking histidine (H) indicates a positive interaction between the bait and prey proteins, and growth with 3-amino-1,2,4-triazole indicates stronger interactions.

### Melanin quantification

For pigment determination, cells were grown in T75 flasks with MNT1 media for 72 h. Cells were washed and trypsinized with 0.05% trypsin, 0.53 mM EDTA, antitrypsinized with MNT1 media, and counted with a Neubauer chamber (Hausser Scientific). About 10^6^ cells were pelleted in 1.7 ml screw cap tubes at 200*g* for 5 min at 4 °C. Pellets were resuspended in 500 μl ice-cold PBS with 1× protease inhibitor cocktail, and 50 μl of 0.5 mm glass beads (Biospec) were added to each tube. Cells were homogenized using a Beadbug-6 homogenizer set to 2500 RPM with three cycles of 30 s on and 30 s off. Tubes were then centrifuged at 16,000*g* at 4 °C for 15 min to pellet melanin. Supernatants containing soluble protein were transferred to separate tubes and used for total protein content determination by Bio-Rad Protein Assay. Melanin pellets were washed by adding 500 μl 100% ethanol and vortexing for 5 s. Melanin was pelleted again by centrifuging at 16,000*g* at 4 °C for 5 min. The ethanol supernatant was aspirated, and residual ethanol was evaporated by incubating open tubes at 37 °C for 30 min. After drying the pellets, 1000 μl freshly diluted 10% (v/v) dimethyl sulfoxide and 1 N NaOH in PBS was added to each pellet and vortexed to resuspend the melanin and incubated for 1 h at 80 °C to solubilize melanin. Tubs were vortexed again 10 min into the incubation and again at 60 min to keep melanin suspended. Meanwhile, *Sepia officinalis* melanin was suspended in 10% (v/v) dimethyl sulfoxide, 1 N NaOH in PBS, diluted in series to generate a standard curve, and incubated at 80 °C for 1 h with other melanin samples. After the incubation, tubes were incubated at room temperature for 20 min and centrifuged at 16,000*g* for 10 min at room temperature to pellet insoluble material. Supernatants were transferred to 1 ml cuvettes with 1 cm path length, and absorbance was measured at 490 nm using a NanoDrop One spectrophotometer (Thermo Scientific). The linear *S. officinalis* standard curve was used to calculate melanin concentration in each melanocyte sample.

### Statistical analysis

Statistical analysis was performed in GraphPad Prism. Sample distributions were tested for normality using the Shapiro–Wilk test. For datasets with two populations, a Student’s *t* test (Fig. 8*B*) or Mann–Whitney test (Figs. 1*B* and 3*B*) was performed as parametric test or nonparametric test, respectively. For datasets with three or more populations, one-way ANOVA with post hoc Tukey test (Figure 4, Figure 6, Figure 7, Figure 9, *B*, *D*, *F*, and *H*) or Kruskal–Wallis test (Figs. 5*D* and 6*F*) was used as parametric test or nonparametric test, respectively. N values and error bars are described in each figure caption. Significance is depicted as ∗*p* ≤ 0.05; ∗∗*p* ≤ 0.01; ∗∗∗*p* ≤ 0.001; and ∗∗∗∗*p* ≤ 0.0001.

## Data availability

All data are included in the article.

## Supporting information

This article contains supporting information.

## Conflict of interest

The authors declare that they have no conflicts of interest with the contents of this article.

## References

[1] Federico J.R., Krishnamurthy K. (2019).

[2] Grønskov K., Ek J., Brondum-Nielsen K. (2007). Oculocutaneous albinism. Orphanet. J. Rare Diseases.

[3] Marti A., Lasseaux E., Ezzedine K., Léauté-Labrèze C., Boralevi F., Paya C. (2018). Lessons of a day hospital: comprehensive assessment of patients with albinism in a European setting. Pigment Cell Melanoma Res..

[4] Pavan W.J., Sturm R.A. (2019). The genetics of human skin and hair pigmentation. Annu. Rev. Genomics Hum. Genet..

[5] Montoliu L., Grønskov K., Wei A.-H., Martínez-García M., Fernández A., Arveiler B. (2014). Increasing the complexity: new genes and new types of albinism. Pigment Cell Melanoma Res..

[6] Bellono N.W., Escobar I.E., Lefkovith A.J., Marks M.S., Oancea E. (2014). An intracellular anion channel critical for pigmentation. eLife.

[7] Garrido G., Fernández A., Montoliu L. (2021). HPS11 and OCA8: two new types of albinism associated with mutations in BLOC1S5 and DCT genes. Pigment Cell Melanoma Res..

[8] K B., Purohit R. (2013). Mutational analysis of TYR gene and its structural consequences in OCA1A. Gene.

[9] Kausar T., Bhatti M., Ali M., Shaikh R., Ahmed Z. (2013). OCA5, a novel locus for non-syndromic oculocutaneous albinism, maps to chromosome 4q24. Clin. Genet..

[10] Le L., Escobar I.E., Ho T., Lefkovith A.J., Latteri E., Haltaufderhyde K.D. (2020). SLC45A2 protein stability and regulation of melanosome pH determine melanocyte pigmentation. Mol. Biol. Cell.

[11] Pennamen P., Tingaud-Sequeira A., Gazova I., Keighren M., McKie L., Marlin S. (2020). Dopachrome tautomerase variants in patients with oculocutaneous albinism. bioRxiv.

[12] Suzuki T., Tomita Y. (2008). Recent advances in genetic analyses of oculocutaneous albinism types 2 and 4. J. Dermatol. Sci..

[13] Wei A.H., Zang D.J., Zhang Z., Liu X.Z., He X., Yang L. (2013). Exome sequencing identifies SLC24A5 as a candidate gene for nonsyndromic oculocutaneous albinism. J. Invest. Dermatol..

[14] Zhang Z., Gong J., Sviderskaya E.V., Wei A., Li W. (2019). Mitochondrial NCKX5 regulates melanosomal biogenesis and pigment production. J. Cell Sci..

[15] Grønskov K., Dooley C.M., Østergaard E., Kelsh R.N., Hansen L., Levesque M.P. (2013). Mutations in c10orf11, a melanocyte-differentiation gene, cause autosomal-recessive albinism. Am. J. Human Genet..

[16] Kamaraj B., Purohit R. (2014). Mutational analysis of oculocutaneous albinism: a compact review. BioMed Res. Inter..

[17] Peng F., Zhu G., Hysi P.G., Eller R.J., Chen Y., Li Y. (2019). Genome-wide association studies identify multiple genetic loci influencing eyebrow color variation in Europeans. J. Invest. Dermatol..

[18] Raposo G., Marks M.S. (2007). Melanosomes--dark organelles enlighten endosomal membrane transport. Nat. Rev. Mol. Cell Biol..

[19] van Niel G., Charrin S., Simoes S., Romao M., Rochin L., Saftig P. (2011). The tetraspanin CD63 regulates ESCRT-independent and -dependent endosomal sorting during melanogenesis. Dev. Cell.

[20] Bissig C., Croisé P., Heiligenstein X., Hurbain I., Lenk G.M., Kaufman E. (2019). The PIKfyve complex regulates the early melanosome homeostasis required for physiological amyloid formation. J. Cell Sci..

[21] van Niel G., Bergam P., Di Cicco A., Hurbain I., Lo Cicero A., Dingli F. (2015). Apolipoprotein E regulates amyloid formation within endosomes of pigment cells. Cell Rep..

[22] Berson J.F., Harper D.C., Tenza D., Raposo G., Marks M.S. (2001). Pmel17 initiates premelanosome morphogenesis within multivesicular bodies. Mol. Biol. Cell.

[23] Ambrosio A.L., Boyle J.A., Aradi A.E., Christian K.A., Di Pietro S.M. (2016). TPC2 controls pigmentation by regulating melanosome pH and size. Proc. Nat. Acad. Sci. U. S. A..

[24] Bellono N.W., Escobar I.E., Oancea E. (2016). A melanosomal two-pore sodium channel regulates pigmentation. Sci. Rep..

[25] Luck K., Kim D.K., Lambourne L., Spirohn K., Begg B.E., Bian W. (2020). A reference map of the human binary protein interactome. Nature.

[26] Bultema J.J., Ambrosio A.L., Burek C.L., Di Pietro S.M. (2012). BLOC-2, AP-3, and AP-1 proteins function in concert with Rab38 and Rab32 proteins to mediate protein trafficking to lysosome-related organelles. J. Biol. Chem..

[27] Bultema J.J., Boyle J.A., Malenke P.B., Martin F.E., Dell'Angelica E.C., Cheney R.E. (2014). Myosin vc interacts with Rab32 and Rab38 proteins and works in the biogenesis and secretion of melanosomes. J. Biol. Chem..

[28] Tamura K., Ohbayashi N., Maruta Y., Kanno E., Itoh T., Fukuda M. (2009). Varp is a novel Rab32/38-binding protein that regulates Tyrp1 trafficking in melanocytes. Mol. Biol. Cell.

[29] Mukherjee D., Gao M., O'Connor J.P., Raijmakers R., Pruijn G., Lutz C.S. (2002). The mammalian exosome mediates the efficient degradation of mRNAs that contain AU-rich elements. EMBO J..

[30] Okuda E.K., Gonzales-Zubiate F.A., Gadal O., Oliveira C.C. (2020). Nucleolar localization of the yeast RNA exosome subunit Rrp44 hints at early pre-rRNA processing as its main function. J. Biol. Chem..

[31] McGrath E., Waschbüsch D., Baker B.M., Khan A.R. (2019). LRRK2 binds to the Rab32 subfamily in a GTP-dependent manner *via* its armadillo domain in. Small GTPases.

[32] Pylypenko O., Hammich H., Yu I.M., Houdusse A. (2018). Rab GTPases and their interacting protein partners: structural insights into Rab functional diversity. Small GTPases.

[33] Tamura K., Ohbayashi N., Ishibashi K., Fukuda M. (2011). Structure-function analysis of VPS9-ankyrin-repeat protein (Varp) in the trafficking of tyrosinase-related protein 1 in melanocytes. J. Biol. Chem..

[34] Kiliç M., Hazar Özcan M., Taskin E., Yildirim H., Şen A. (2021). Oculocutaneous albinism type 7 with recurrent infections: a case report. Asthma Allergy Immunol..

[35] Harper D.C., Theos A.C., Herman K.E., Tenza D., Raposo G., Marks M.S. (2008). Premelanosome amyloid-like fibrils are composed of only Golgi-processed forms of Pmel17 that have been proteolytically processed in endosomes. J. Biol. Chem..

[36] Watt B., van Niel G., Fowler D.M., Hurbain I., Luk K.C., Stayrook S.E. (2009). N-terminal domains elicit formation of functional Pmel17 amyloid fibrils. J. Biol. Chem..

[37] Mitchell S.M., Graham M., Liu X., Leonhardt R.M. (2021). Identification of critical amino acid residues in the regulatory N-terminal domain of PMEL. Sci. Rep..

[38] Hee J.S., Mitchell S.M., Liu X., Leonhardt R.M. (2017). Melanosomal formation of PMEL core amyloid is driven by aromatic residues. Sci. Rep..

[39] Graham M., Tzika A.C., Mitchell S.M., Liu X., Leonhardt R.M. (2019). Repeat domain-associated O-glycans govern PMEL fibrillar sheet architecture. Sci. Rep..

[40] Sires-Campos J., Lambertos A., Delevoye C., Raposo G., Bennett D.C., Sviderskaya E. (2021). Mahogunin Ring Finger 1 regulates pigmentation by controlling the pH of melanosomes in melanocytes and melanoma cells. Cell Mol. Life Sci..

[41] Ancans J., Tobin D.J., Hoogduijn M.J., Smit N.P., Wakamatsu K., Thody A.J. (2001). Melanosomal pH controls rate of melanogenesis, eumelanin/phaeomelanin ratio and melanosome maturation in melanocytes and melanoma cells. Exp. Cell Res..

[42] Wada S., Hamada M., Kobayashi K., Satoh N. (2008). Novel genes involved in canonical Wnt/β-catenin signaling pathway in early Ciona intestinalis embryos. Dev. Growth Differ..

[43] Berson J.F., Theos A.C., Harper D.C., Tenza D., Raposo G., Marks M.S. (2003). Proprotein convertase cleavage liberates a fibrillogenic fragment of a resident glycoprotein to initiate melanosome biogenesis. J. Cell Biol..

[44] Rochin L., Hurbain I., Serneels L., Fort C., Watt B., Leblanc P. (2013). BACE2 processes PMEL to form the melanosome amyloid matrix in pigment cells. Proc. Natl. Acad. Sci. U. S. A..

[45] Hellström A.R., Watt B., Fard S.S., Tenza D., Mannström P., Narfström K. (2011). Inactivation of Pmel alters melanosome shape but has only a subtle effect on visible pigmentation. PLoS Genet..

[46] Lahola-Chomiak A.A., Footz T., Nguyen-Phuoc K., Neil G.J., Fan B., Allen K.F. (2019). Non-Synonymous variants in premelanosome protein (PMEL) cause ocular pigment dispersion and pigmentary glaucoma. Hum. Mol. Genet..

[47] Gerondopoulos A., Langemeyer L., Liang J.-R., Linford A., Barr F.A. (2012). BLOC-3 mutated in Hermansky-Pudlak syndrome is a Rab32/38 guanine nucleotide exchange factor. Current Biol..

[48] Uhlen M., Fagerberg L., Hallstrom B.M., Lindskog C., Oksvold P., Mardinoglu A. (2015). Proteomics. Tissue-based map of the human proteome. Science.

[49] Hu Z.-q., Rao C.-l., Tang M.-l., zhang Y., Lu X.-x., Chen J.-g. (2019). Rab32 GTPase, as a direct target of miR-30b/c, controls the intracellular survival of Burkholderia pseudomallei by regulating phagosome maturation. PLoS Pathog..

[50] Baldassarre M., Solano-Collado V., Balci A., Colamarino R.A., Dambuza I.M., Reid D.M. (2021). The Rab32/BLOC-3-dependent pathway mediates host defense against different pathogens in human macrophages. Sci Adv..

[51] Drizyte-Miller K., Chen J., Cao H., Schott M.B., McNiven M.A. (2020). The small GTPase Rab32 resides on lysosomes to regulate mTORC1 signaling. J. Cell Sci..

[52] Ambrosio A.L., Boyle J.A., Di Pietro S.M. (2012). Mechanism of platelet dense granule biogenesis: Study of cargo transport and function of Rab32 and Rab38 in a model system. Blood.

[53] Aguilar A., Weber J., Boscher J., Freund M., Ziessel C., Eckly A. (2019). Combined deficiency of RAB32 and RAB38 in the mouse mimics Hermansky-Pudlak syndrome and critically impairs thrombosis. Blood Adv..

[54] Thibord F., Chan M.V., Chen M.H., Johnson A.D. (2022). A year of Covid-19 GWAS results from the GRASP portal reveals potential genetic risk factors. HGG Adv..

[55] Keramati A.R., Chen M.H., Rodriguez B.A.T., Yanek L.R., Bhan A., Gaynor B.J. (2021). Genome sequencing unveils a regulatory landscape of platelet reactivity. Nat. Commun..

[56] Nakamura S., Takayama N., Hirata S., Seo H., Endo H., Ochi K. (2014). Expandable megakaryocyte cell lines enable clinically applicable generation of platelets from human induced pluripotent stem cells. Cell Stem Cell.

[57] Ito Y., Nakamura S., Sugimoto N., Shigemori T., Kato Y., Ohno M. (2018). Turbulence activates platelet biogenesis to enable clinical scale *ex vivo* production. Cell.

[58] Ambrosio A.L., Febvre H.P., Di Pietro S.M. (2022). Syntaxin 12 and COMMD3 are new factors that function with VPS33B in the biogenesis of platelet alpha-granules. Blood.

[59] Huizing M., Helip-Wooley A., Westbroek W., Gunay-Aygun M., Gahl W.A. (2008). Disorders of lysosome-related organelle biogenesis: clinical and molecular genetics. Annu. Rev. Genomics Hum. Genet..

[60] Dennis M.K., Delevoye C., Acosta-Ruiz A., Hurbain I., Romao M., Hesketh G.G. (2016). BLOC-1 and BLOC-3 regulate VAMP7 cycling to and from melanosomes *via* distinct tubular transport carriers. J. Cell Biol..

[61] Marubashi S., Shimada H., Fukuda M., Ohbayashi N. (2016). RUTBC1 functions as a GTPase-activating protein for Rab32/38 and regulates melanogenic enzyme trafficking in melanocytes. J. Biol. Chem..

[62] Ambrosio A.L., Di Pietro S.M. (2019). Mechanism of platelet α-granule biogenesis: study of cargo transport and the VPS33B-VPS16B complex in a model system. Blood Adv..

[63] Zewe J.P., Miller A.M., Sangappa S., Wills R.C., Goulden B.D., Hammond G.R.V. (2020). Probing the subcellular distribution of phosphatidylinositol reveals a surprising lack at the plasma membrane. J. Cell Biol..

[64] Ambrosio A.L., Boyle J.A., Di Pietro S.M. (2015). TPC2 mediates new mechanisms of platelet dense granule membrane dynamics through regulation of Ca2+ release. Mol. Biol. Cell.

[65] Giddings T.H., Morphew M.K., McIntosh J.R. (2017). Preparing fission yeast for electron microscopy. Cold Spring Harb. Protoc..

[66] Di Pietro S.M., Cascio D., Feliciano D., Bowie J.U., Payne G.S. (2010). Regulation of clathrin adaptor function in endocytosis: novel role for the SAM domain. EMBO J..

[67] Gietz R.D., Schiestl R.H. (2007). Frozen competent yeast cells that can be transformed with high efficiency using the LiAc/SS carrier DNA/PEG method. Nat. Protoc..

[68] Starcevic M., Dell'Angelica E.C. (2004). Identification of snapin and three novel proteins (BLOS1, BLOS2, and BLOS3/reduced pigmentation) as subunits of biogenesis of lysosome-related organelles complex-1 (BLOC-1). J. Biol. Chem..

[69] Kelley L.A., Mezulis S., Yates C.M., Wass M.N., Sternberg M.J. (2015). The Phyre2 web portal for protein modeling, prediction and analysis. Nat. Protoc..

