# Supplementary material for: OCA7 is a melanosome membrane protein that defines pigmentation by regulating early stages of melanosome biogenesis
Source: J Biol Chem. 2022 Nov 9;298(12):102669. doi: 10.1016/j.jbc.2022.102669 (PMC9703636; doi:10.1016/j.jbc.2022.102669)
Supplement: Supporting information [file mmc1.pdf]

Supporting Information

OCA7 is a melanosome membrane protein that defines pigmentation by regulating early stages of melanosome biogenesis

Wyatt C. Beyers, Anna M. Detry and Santiago M. Di Pietro

List of Supporting Information

Figure S1. OCA7-EGFP colocalizes with melanosome markers.

Figure S2. mCherry-CD63 recovers slowly after photobleaching.

Figure S3. Rab38 membrane localization is sufficient for OCA7 recruitment to membranes.

Figure S4. Validation of OCA7-KO MNT1 cells and anti-OCA7 antibodies.

Figure S5. The steady state levels of melanogenic enzymes are unaffected in OCA7-KO MNT1 cells.

Figure S6. OCA7 functions independently of TPC2.

Figure S7. OCA7 is expressed in multiple cell types.

Table S1. Primers for Cloning.

Table S2. OCA7 Knockout Predesigned crRNA.

Table S3. OCA7 KO Genotyping Primers.

Figure S1

Beyers et al.

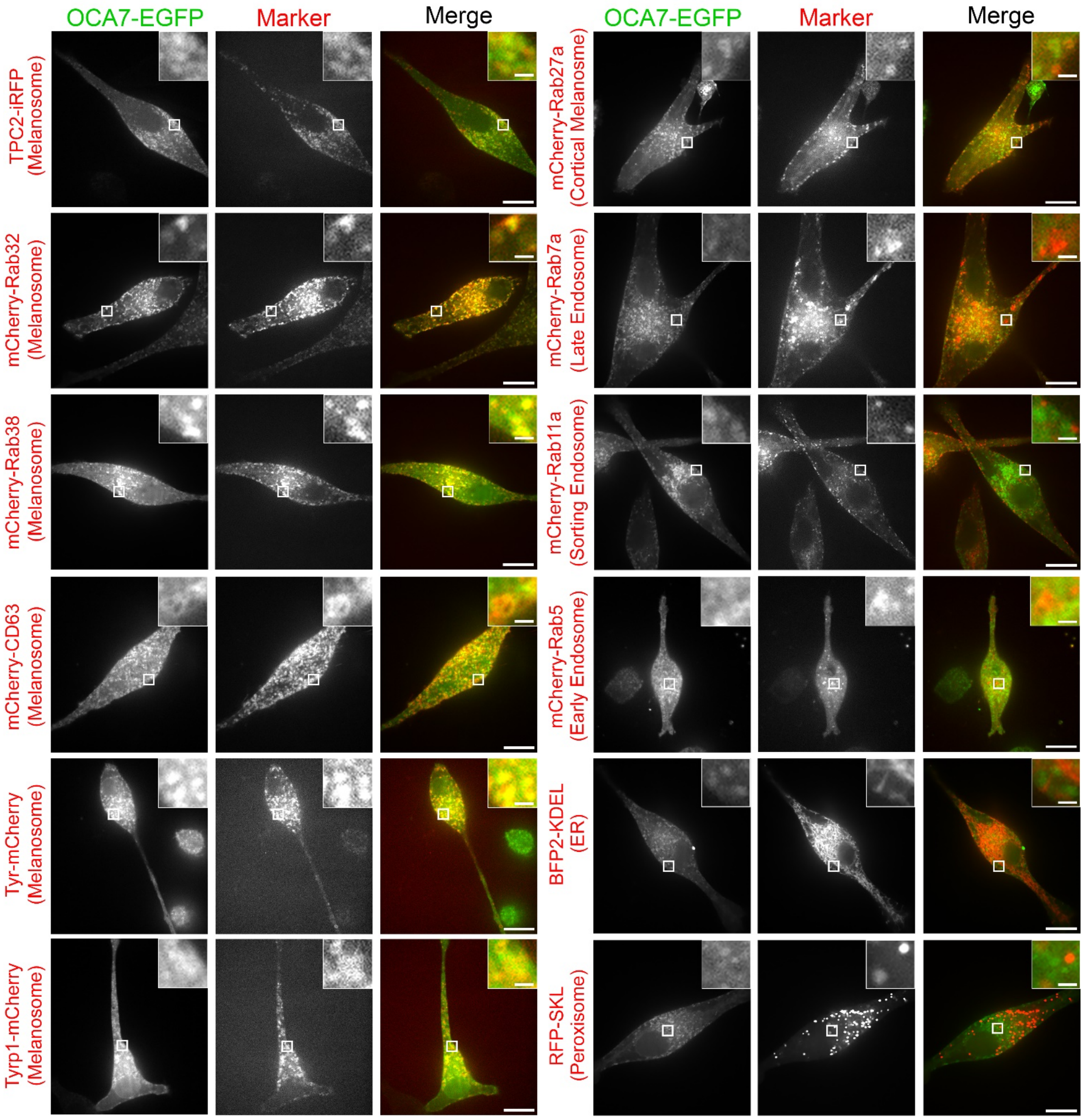

Figure S1. OCA7-EGFP colocalizes with melanosome markers. Live cell confocal fluorescence images of MNT1 cells expressing OCA7-EGFP (green) with melanosome markers or various other organelle markers (red). Melanosome markers include TPC2-iRFP, mCherry-Rab32, mCherry-Rab38, mCherry-CD63, Tyr-mCherry, and Tyrp1-mCherry. Other markers include mCherry-Rab27a for stage IV cortical melanosomes, mCherry-Rab7a for late endosomes/lysosomes, mCherry-Rab11a for sorting/recycling endosomes, mCherry-

Rab5a for early endosomes, BFP2-KDEL for endoplasmic reticulum and RFP-SKL for peroxisomes. Insets highlight regions where OCA7 is on the same or separate organelles as the marker. Scale bars indicate 10μm for unmagnified images and 1μm for magnified insets.

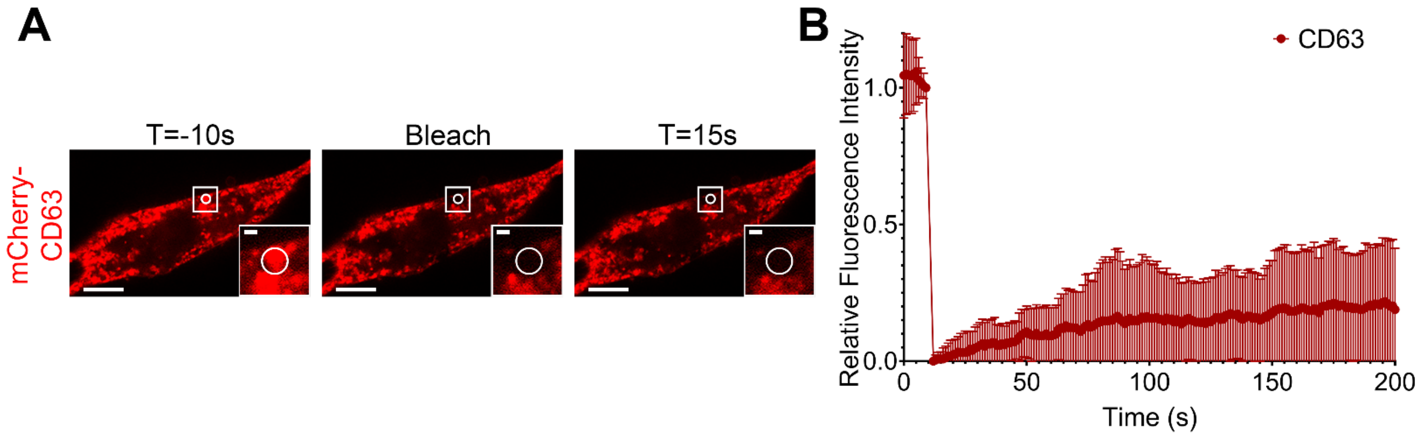

Figure S2. mCherry-CD63 recovers slowly after photobleaching. (A) FRAP experiment with MNT1 cells expressing mCherry-CD63, a melanosome localized integral membrane protein, showing slow recovery after photobleaching. Images are shown for timepoints 10 sec before photobleaching, immediately after, and 15 sec after. Circles in the insets indicate melanosomes analyzed by FRAP. Scale bars indicate 10μm for unmagnified images and 1μm for magnified insets. **(B)** Quantification of FRAP time lapses shown in (A) depicting mean fluorescence signal relative to the timepoint immediately before photobleaching. Error bars represent S.D. (200 timepoints were measured for n=31 melanosomes imaged in 12 cells).

Figure S3 Beyers et al.

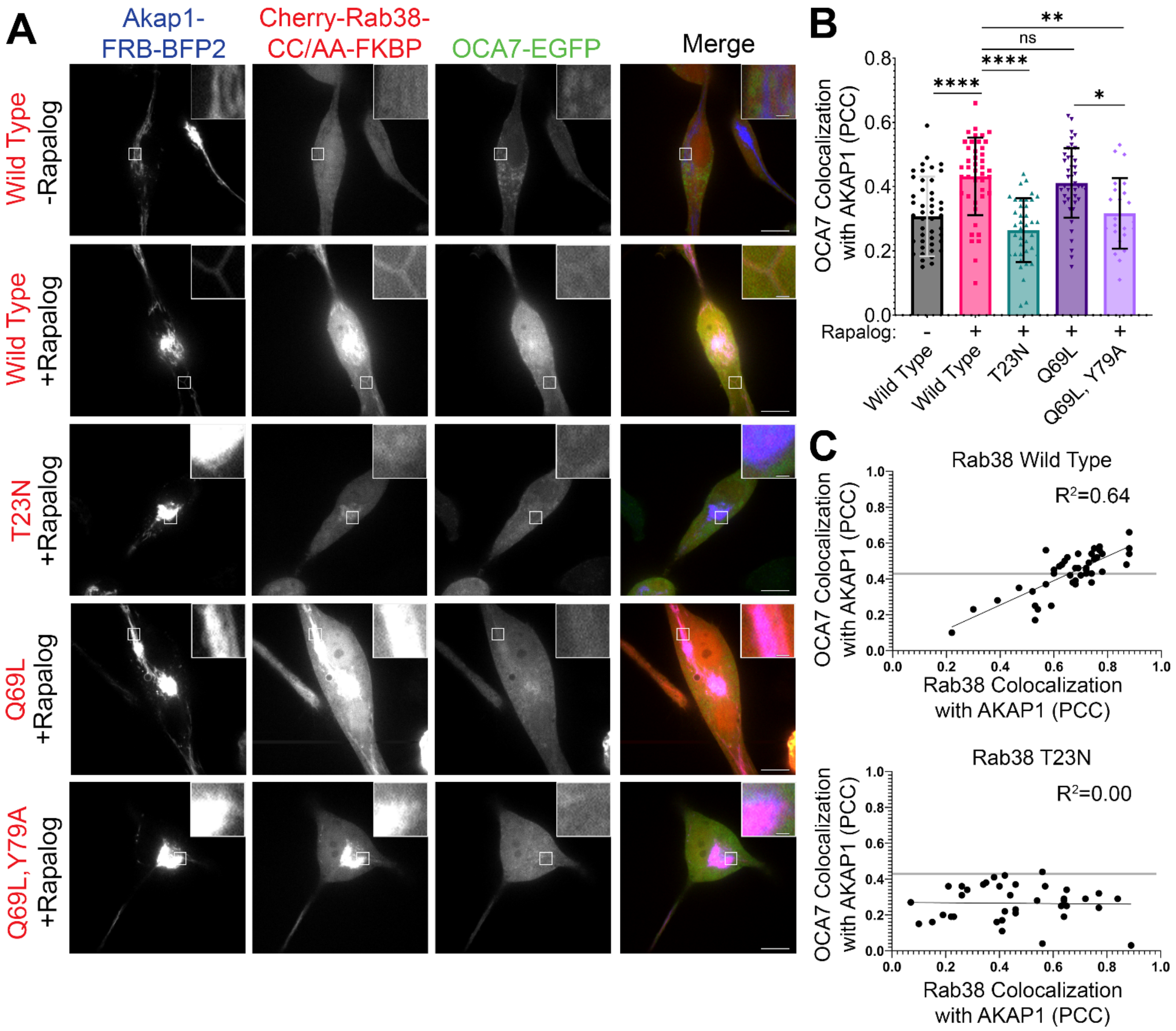

Figure S3. Rab38 membrane localization is sufficient for OCA7 recruitment to membranes. (A) Spinning disc confocal fluorescence microscopy images of MNT1 cells expressing OCA7-EGFP, Akap1-FRB-BFP2, and mCherry-Rab38-CC/AA-FKBP (wild type or various mutants) in the absence or presence of 100nM Rapalog. In the absence of Rapalog, mCherry-Rab38-CC/AA-FKBP has a cytosolic distribution and OCA7-EGFP has a cytosolic or punctate (melanosome) distribution. Upon addition of 100nM Rapalog, mCherry-Rab38-CC/AA-FKBP (wild type and various mutants) relocalizes to Akap1-FRB-BFP2 labeled mitochondria. Concomitantly, OCA7-EGFP mislocalizes to mitochondria with Wild Type or GTP-locked, constitutively active mutant (Q69L) mCherry-Rab38-CC/AA-FKBP but not the GDP-locked, dominant negative mutant (T23N). Expression of the

corresponding Rab38 Q69L, Y79A double mutant (Q69L, Y79A), which is constitutively active but unable to bind OCA7 shows reduced OCA7-EGFP recruitment to mitochondria compared to the Rab38 Q69L constitutively active mutant. Insets show regions where mitochondria are visible (Akap1-FRB-BFP2 channel). Scale bars indicate 10μm for unmagnified images and 1μm for magnified insets. **(B)** Graph of PCC for images in (A) depicting OCA7-EGFP colocalization with Akap1-FRB-BFP2, indicating mislocalization to mitochondria. The graph shows mean ± S.D. n=46, n=43, n=39, n=43, and n=23 cells were analyzed for WT -Rapalog, WT +Rapalog, T23N +Rapalog, Q69L +Rapalog, and Q69L, Y79A +Rapalog, respectively. Statistical significance was tested by One way ANOVA and post-hoc Tukey tests. For statistical comparisons, ns, *,**, and **** correspond to $p>0.05$, $p<0.05$, $p<0.01$, and $p<0.0001$ respectively. **(C)** Regression analysis investigating the correlation between mCherry-Rab38-CC/AA-FKBP / Akap1-FRB-BFP2 dimerization efficiency (Rab38 colocalization with AKAP1, PCC) and OCA7-EGFP recruitment to mitochondria (OCA7 colocalization with AKAP1, PCC). OCA7-EGFP recruitment to mitochondria correlated highly with dimerization efficiency when the Rab38 Wild Type form of mCherry-Rab38-CC/AA-FKBP was expressed but not when the corresponding Rab38 T23N dominant negative mutant was expressed. For comparison, the horizontal line in both graphs represents the mean PCC for OCA7-EGFP colocalization with Akap1-FRB-BFP2 for cells expressing Wild Type Rab38 as a reference.

Figure S4

Beyers et al.

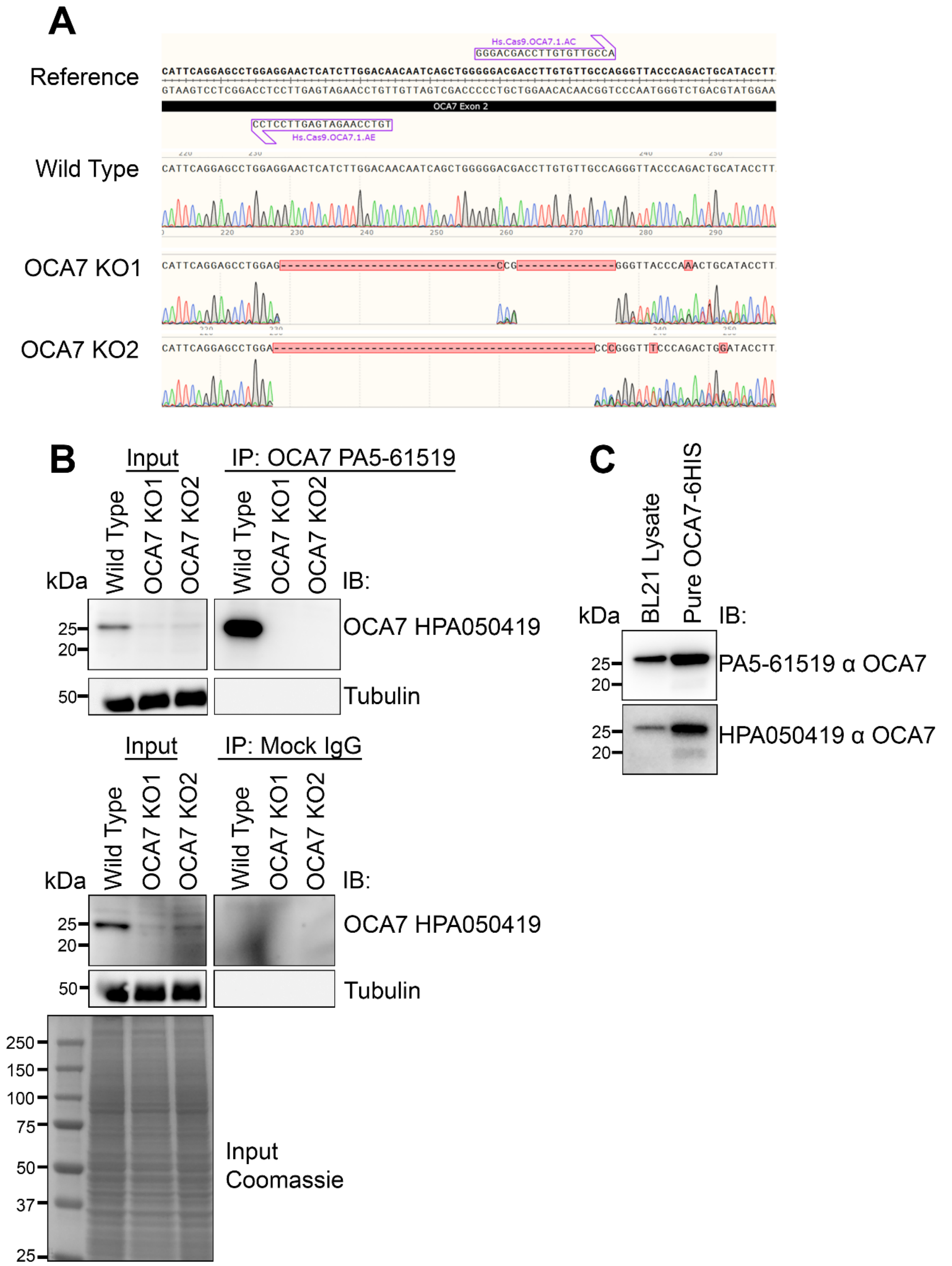

Figure S4. Validation of OCA7-KO MNT1 cells and anti-OCA7 antibodies. (A) Sequencing analysis of OCA7 PCR product amplified from genomic DNA obtained from Wild Type or OCA7-KO MNT1 cell lines. For the OCA7-KO cell lines, red highlighted dashes indicate sequence that was successfully excised via CRISPR-Cas9 and

overlapping peaks likely represent indels. **(B)** Immunoblotting (IB) analysis of TX100 cell lysate input and anti-OCA7 or Mock (IgG) immunoprecipitation samples obtained from Wild Type or two different clones of OCA7-KO MNT1 cells used in the study. Samples were immunoprecipitated (IP) with Rabbit anti-OCA7 (PA5-61519) or total rabbit IgG and the immunoblot was probed with a different Rabbit anti-OCA7 (HPA050419) antibody showing specificity for endogenous OCA7. The immunoblots of the IP samples indicate that OCA7 is absent from the OCA7-KO cell lysates and residual signal in the input is likely background. Even gel loading is depicted by Tubulin immunoblots and the corresponding Coomassie stained gel. **(C)** Immunoblot of recombinantly expressed OCA7-6His showing both antibodies tested detect OCA7-6His in BL21 bacteria cell lysate or in its pure form.

Figure S5 Beyers et al.

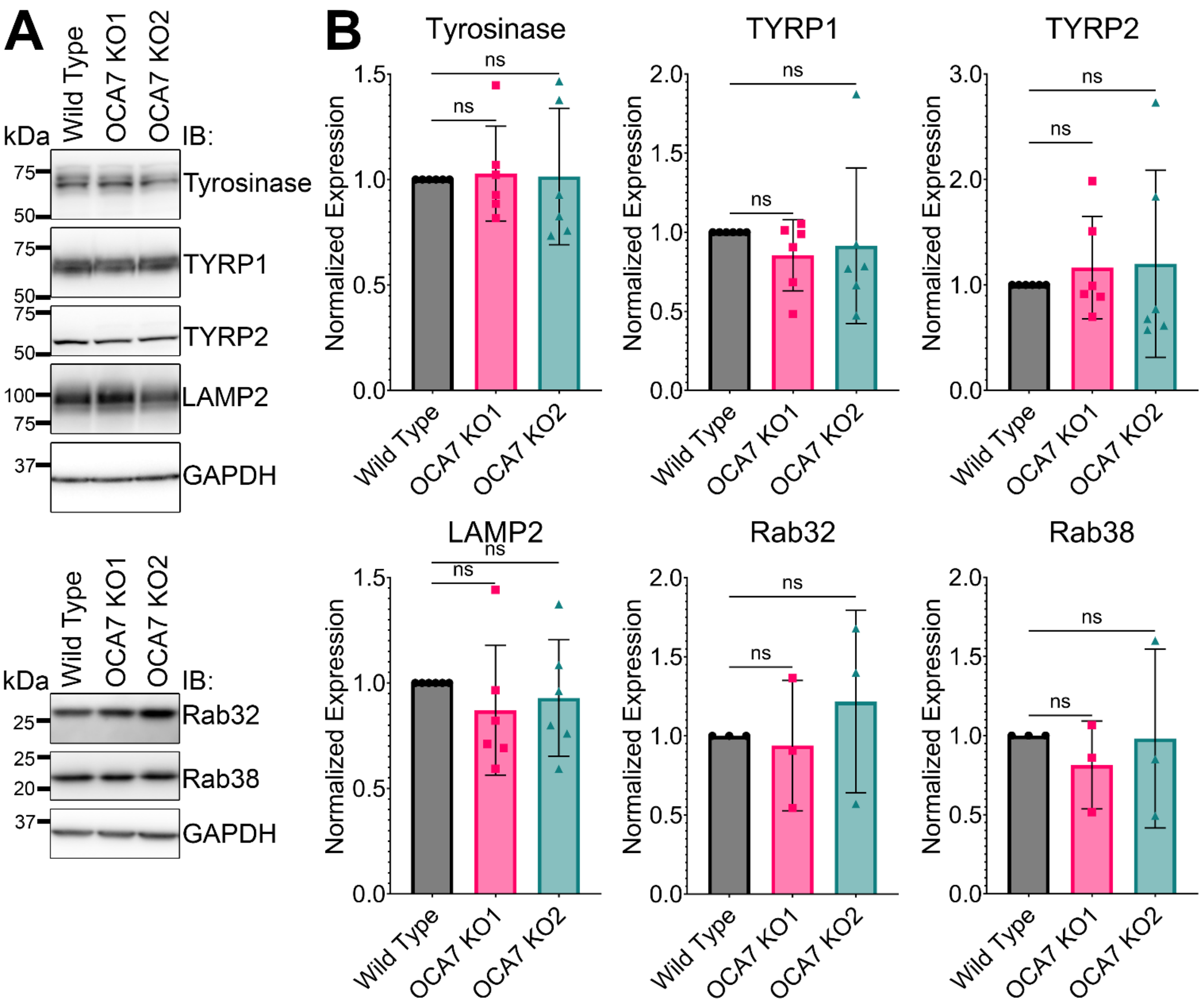

Figure S5. The steady state levels of melanogenic enzymes are unaffected in OCA7-KO MNT1 cells.

(A) Immunoblots of RIPA lysates from Wild Type or OCA7-KO MNT1 cells probed with antibodies for Tyrosinase, TYRP1, TYRP2, and other lysosome/late endosome or melanosome proteins LAMP2, Rab32, Rab38, and GAPDH as a loading control. Immunoblots show expression of melanogenic enzymes is unaffected in OCA7-KO cells. **(B)** Quantification of immunoblots shown in (A). The graphs show mean ± S.D and are shown relative to Wild Type. Immunoblots were quantified using RIPA lysates from n=6 independent experiments for Tyrosinase, TYRP1, TYRP2 and LAMP2, and n=3 independent experiments for Rab32 and Rab38. Statistical significance was tested using ordinary one way ANOVA and Tukey post-hoc tests. ns corresponds to p>0.05.

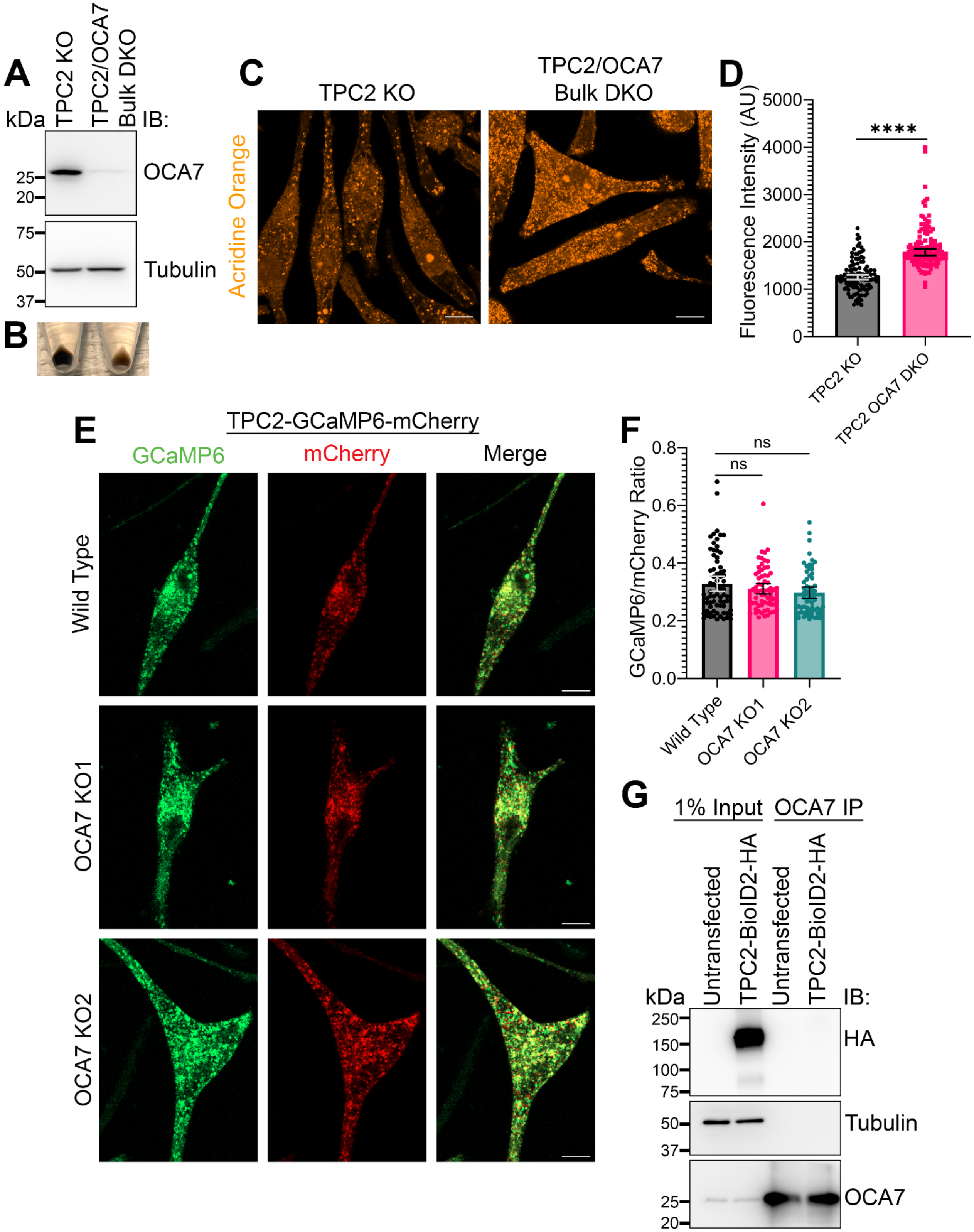

Figure S6. OCA7 functions independently of TPC2. (A) Immunoblot of RIPA lysates obtained from parental TPC2-KO MNT1 cells and bulk TPC2/OCA7 double-KO MNT1 cells indicating efficient reduction of OCA7 protein

expression. **(B)** Images of parental TPC2-KO MNT1 and bulk TPC2/OCA7 double-KO MNT1 cell pellets showing less pigmentation in the double-KO cells relative to TPC2-KO cells. Cell pellets correspond to one 10cm dish approximately 70% confluent. **(C)** Live cell laser scanning Airyscan SR fluorescence microscopy images of TPC2-KO MNT1 and bulk TPC2/OCA7 double-KO MNT1 cells stained with Acridine Orange for 30 minutes showing heightened Acridine Orange accumulation in the double-KO cells. Scale bars indicate 10μm. **(D)** Quantification of Acridine Orange fluorescence intensity for cells from (C). The graph shows median with error bars showing the 95% confidence interval. n=119 and n=128 cells were analyzed for TPC2-KO and TPC2/OCA7 double-KO respectively. Significance was tested using the Mann-Whitney test and **** corresponds to $p<0.0001$ **(E)** Live cell laser scanning Airyscan SR fluorescence microscopy images of WT and OCA7-KO MNT1 cells transiently expressing the calcium sensor TPC2-GCaMP6-Cherry. Cells were imaged 24 hours after transfection. Scale bars indicate 10μm. **(F)** Quantification of GCaMP6/mCherry fluorescence intensity ratio for cells imaged in (E) indicating no difference in basal calcium in the melanosome periphery. The graph shows the medians with 95% confidence intervals. n=70, n=66, and n=63 cells were analyzed for WT, OCA7-KO1 and OCA7-KO2 respectively. Significance was tested using the Kruskal-Wallis test. n.s corresponds to $p>0.05$ **(G)** Immunoblot analysis of TX100 cell lysate input and anti-OCA7 immunoprecipitation of lysates derived from untransfected MNT1 cells or MNT1 cells overexpressing TPC2-BioID2-HA. The immunoblot indicates overexpressed TPC2 does not co-immunoprecipitate with endogenous OCA7.

Figure S7

Beyers et al.

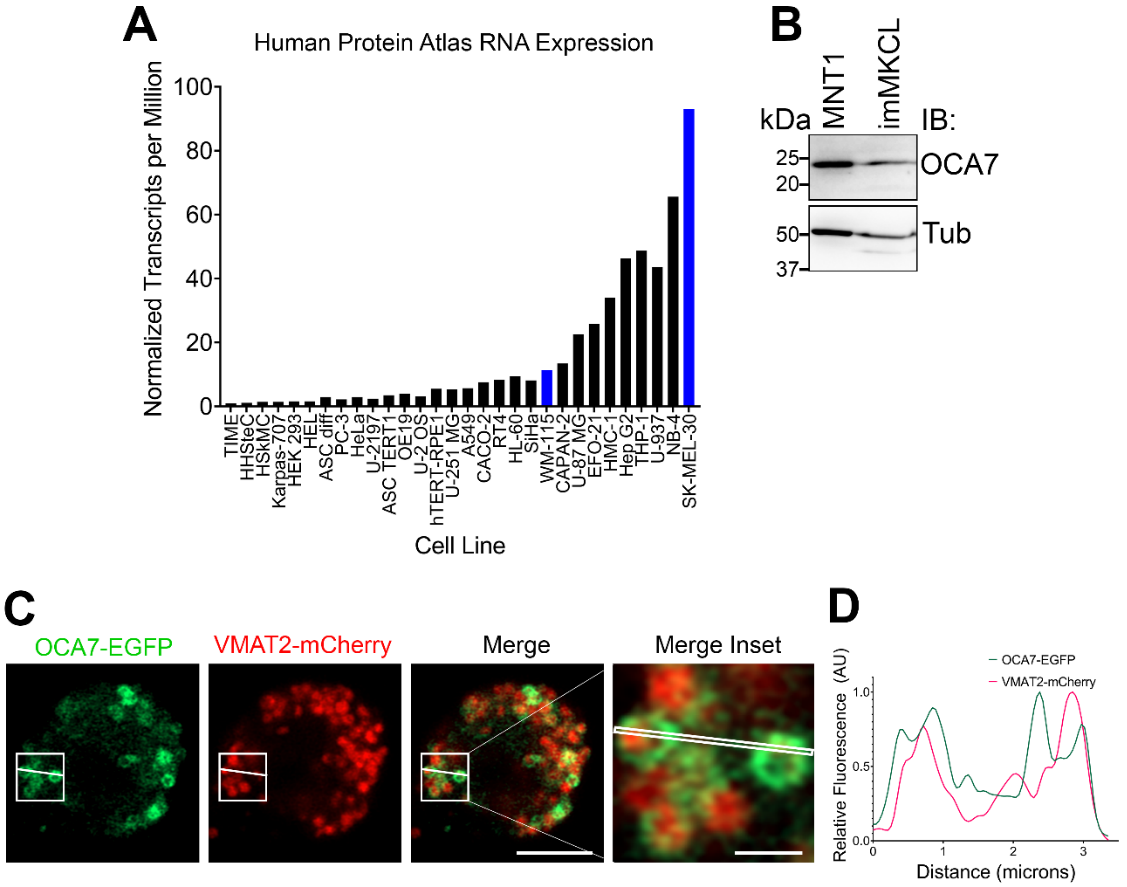

Figure S7. OCA7 is expressed in multiple cell types. (A) Graph of OCA7 RNA expression showing the OCA7 gene is expressed in several cell types in addition to melanocytes. The blue bars indicate melanoma cell lines. OCA7 expression data was downloaded from the Human Protein Atlas database and 30 common cell lines are shown. (proteinatlas.org/ENSG00000148655-LRMDA/subcellular). **(B)** Immunoblot of RIPA lysates obtained from MNT1 cells or imMKCL megakaryocytes showing OCA7 is expressed. **(C)** Laser scanning Airyscan SR fluorescence microscopy images of imMKCL cells differentiated for 72 hours expressing OCA7-EGFP and VMAT2-mCherry. OCA7-EGFP is present in some overlapping compartments with VMAT2-mCherry suggesting partial dense granule localization. Scale bars indicate 5µm for the unmagnified image and 1µm for magnified inset. **(D)** Line scan analysis of the indicated region in (C) showing an example of OCA7-EGFP colocalized with VMAT2-mCherry.

Table S1. Primers for Cloning.

Primer Name	Sequence (5'-3')	Plasmid Made
OCA7 Forward	TCTCGAGCTCAAGCTATGGCCGGGCTCGT	pEGFP-N3-OCA7
OCA7 Reverse	TGGCGATGGATCCCGCGAGCTGGTCATCTCGGATAAACCT	pEGFP-N3-OCA7
OCA7 Forward	AAGGAGATATACATATGATGGCCGGGCTCGTGG	pET30(+)-OCA7-6his
OCA7 Reverse	TGCTCGAGTGCGGCCGCGAGCTGGTCATCTCGGA	pET30(+)-OCA7-6his
OCA7 Forward	AAAAGAGATCGAATTGGCCGGGATGGCCGGGCTCGT	pGAD424-OCA7
OCA7 Reverse	ATCTCTGCAGGTCGATCAGAGCTGGTCATCTCGGATAAAC	pGAD424-OCA7
AP1M1 Forward	TGTATCGCCGGAATTCATGTCCGCCAGCGCCGTCTAC	pGBT9-AP1M1
AP1M1 Reverse	TTGGCTGCAGGTCGACTCACTGGGTCCGGAGCTGGTAATCTC	pGBT9-AP1M1
RabIF Forward	TGTATCGCCGGAATTCATGGAACCAGCGGAGCAG	pGBT9-RabIF
RabIF Reverse	TTGGCTGCAGGTCGACTTACTCATGGGAAACTCGTTCCAAGG	pGBT9-RabIF
EXOSC5 Forward	TGTATCGCCGGAATTCATGGAGGAGGAGACGCATACT	pGBT9-EXOSC5
EXOSC5 Reverse	TTGGCTGCAGGTCGACTCAGCTCTTGGAGTAACGCC	pGBT9-EXOSC5
mBFP2 Forward	CAGACTACGCACCGGTTATGGTGTCTAAGGGCGAAGAGC	AKAP1-FRB-BFP2
mBFP2 Reverse	TCTAGAGTCGCGGCCGCTTAATTAAGCTTGTGCCCCAGTTTG	AKAP1-FRB-BFP2
pmCherry-Rab32 CC/AA Mutagenesis Forward	GTCGACGGTACCGCGGGC	pmCherry-C2-Rab32-CC/AA-FKBP
pmCherry-Rab32 CC/AA Mutagenesis Reverse	GGCAGCCTGGGATTTGTTCTCTGCTCTCAAGG	pmCherry-C2-Rab32-CC/AA-FKBP
FKBP12 Forward	AAATCCCAGGCTGCCGGAGTGCAGGTGGAAACCATCTC	pmCherry-C2-Rab32-CC/AA-FKBP
FKBP12 Reverse	CGCGGTACCGTCGACTCATTCCAGTTTTAGAAGCTCCACATCG	pmCherry-C2-Rab32-CC/AA-FKBP
pmCherry-Rab38 CC/AA Mutagenesis Forward	GTCGACGGTACCGCGGGC	pmCherry-C2-Rab38-CC/AA-FKBP
pmCherry-Rab38 CC/AA Mutagenesis Reverse	GGATTTGGCAGCGCCAGAGGCGCTGGCAACCTTG	pmCherry-C2-Rab38-CC/AA-FKBP
FKBP12 Forward	GGCGCTGCCAAATCCGGAGTGCAGGTGGAAACCATCTC	pmCherry-C2-Rab38-CC/AA-FKBP
FKBP12 Reverse	CGCGGTACCGTCGACTCATTCCAGTTTTAGAAGCTCCACATCG	pmCherry-C2-Rab38-CC/AA-FKBP
GCaMP6 C Terminus Forward For addition of restriction site	CGCCACGTGATGACAAACCTTGG	TPC2-GCaMP6-mCherry
GCaMP6 C Terminus Reverse For addition of restriction site	TTTTGCGGCCGCTTTTTACCGGTGATGGCTGATTATGATCTAGAGTCGTGGCCGCTGACTTCGCTGTCATC	TPC2-GCaMP6-mCherry
TPC2 Forward	GCCTGTTAACCGGTCATGGCGGAACCCCAGGC	TPC2-BioID2-HA
TPC2 Reverse	ACTTCCACCGCCTCCAAGCTTCCTGCACAGCCAC	TPC2-BioID2-HA
MCS-13X Linker-BioID2-HA Linearization Forward	GACCGGTTAACAGGCCTTAAGCG	TPC2-BioID2-HA
MCS-13X Linker-BioID2-HA Linearization Reverse	GGAGGCGGTGGAAGTGGAG	TPC2-BioID2-HA

Table S2. OCA7 Knockout Predesigned crRNA.

IDT crRNA	Targeted Sequence in OCA7 Gene (5'-3')
Hs.Cas9.OCA7.1.AC	GGGACGACCTTGTGTTGCCA
Hs.Cas9.OCA7.1.AE	TGTCCAAGATGAGTTCCTCC

Table S3. OCA7 KO Genotyping Primers.

Primer Name	Sequence (5'-3')
Forward	CAATGCGTGAAGGATGCGGT
Reverse	TCTGAAGTGTCCCGAGTGCC
